# LightWaveNet: a lightweight wavelet-enhanced high-low-frequency-aware network with multi-stage supervision for rice disease recognition

**DOI:** 10.3389/fpls.2026.1692649

**Published:** 2026-01-29

**Authors:** Weiqiang Pi, Tao Zhang, Rongyang Wang, Zhongyou Zhou, Guowei Ma, Yong Wang

**Affiliations:** 1 College of Intelligent Manufacturing and Elevator, Huzhou Vocational and Technical College, Huzhou, China; 2 College of Mechanical and Electrical Engineering, Inner Mongolia Agricultural University, Hohhot, China

**Keywords:** deep learning, lightweight model, precision agriculture, rice disease, wavelet convolution

## Abstract

**Introduction:**

Accurate identification of rice diseases is critical for ensuring food security and advancing intelligent agricultural management. However, existing deep learning methods, while achieving high accuracy, often involve heavy computational costs and complex models, which limit their deployment on resource-constrained agricultural devices. More importantly, most of these methods rely on spatial domain representations and cannot model both high- and low-frequency information, making it difficult to capture fine-grained textures and overall structural features of diseased areas simultaneously.

**Methods:**

To address these challenges, this study proposes a lightweight wavelet-enhanced high-low-frequency-aware network (LightWaveNet) for rice disease recognition. Specifically, LightWaveNet employs a parallel structure of wavelet convolution and max pooling to achieve collaborative learning of high- and low-frequency features, enabling effective extraction of both fine-grained textures and overall structural patterns. In the downsampling stage, a parallel design of max pooling and average pooling is adopted to further preserve the complementarity of frequency features. In addition, a multi-stage supervision mechanism is introduced to constrain and optimize features at different levels during training, thereby improving convergence speed and model robustness.

**Results:**

Experimental results demonstrate that LightWaveNet achieves a favorable balance between accuracy and efficiency. With only 0.28 M parameters and 0.02 G floating-point operations (FLOPs), it reaches 95.90% recognition accuracy. Compared with the lightest Mobilenetv2 model among the comparison methods (2.24 M parameters and 0.30 G FLOPs), LightWaveNet exhibits lower computational complexity while achieving higher recognition accuracy.

**Discussion:**

This study provides a feasible solution for rapid rice disease identification and intelligent prevention, while also offering new insights into the design of lightweight recognition networks for agricultural applications.

## 1 Introduction

Rice is one of the most important staple crops worldwide and serves as a cornerstone of global food security (Lv et al., 2025). According to the Food and Agriculture Organization of the United Nations, more than half of the global population relies on rice as a primary food source, providing nearly one-fifth of the total human caloric intake (Liu et al., 2024a). In developing countries, particularly in Asia and Africa, rice is not only a key source of energy and protein but also the foundation of livelihoods for hundreds of millions of people, playing a vital role in the global economy (Chakrabarty et al., 2024; Haikal et al., 2024). With the continuous growth of the world population, ensuring stable and high rice yields is essential to addressing future food challenges and maintaining social stability (Zhao et al., 2025).

However, rice production faces severe challenges from various biotic stresses, with plant diseases being among the most critical threats (Lv et al., 2024). Diseases caused by pathogens such as fungi, bacteria, and viruses, including rice blast and bacterial leaf blight, have led to substantial yield losses worldwide (Jin et al., 2025; Kang et al., 2025). It is estimated that these diseases cause 10% to 30% of annual global rice yield loss, with rates exceeding 50% during severe outbreaks (Nalley et al., 2016; Zhao et al., 2024). Such reductions translate directly into significant economic costs, increasing farmers’ expenditures on fungicides and labor, while also driving up food prices and exacerbating food insecurity in low-income regions (Hu et al., 2025). Therefore, early and accurate identification of rice diseases is essential for implementing precise control strategies, minimizing yield losses, and ensuring global food security.

In the early stages, the diagnosis of rice diseases relied primarily on manual visual inspection by agricultural experts or experienced farmers. Although this approach played a role to some extent, it has several inherent limitations (Song et al., 2024). First, the accuracy of the diagnosis depends heavily on the expertise and knowledge of the individual, making the process highly subjective and prone to misjudgment or omission (Wei et al., 2025). Second, manual diagnosis is time-consuming and labor-intensive, which limits its ability to meet the demands of real-time and continuous monitoring over large-scale farmland (Zhang et al., 2022; Zeng and He, 2024). In addition, professional plant pathologists are scarce and costly. This shortage is particularly severe in remote and underdeveloped regions, where farmers often lack timely access to expert diagnostic support (Quan et al., 2024). Furthermore, the early symptoms of different diseases can be very similar, and sometimes indistinguishable from signs of abiotic stresses such as nutrient deficiencies, which further increases the complexity and uncertainty of manual diagnosis.

With the advancement of computer vision and image processing technologies, traditional machine learning methods have gradually been applied to the detection of early-stage disease images. Examples include support vector machines (SVM), k-nearest neighbors (KNN), random forests (RF), and naive Bayes classifiers (Zhang et al., 2025c; Pan et al., 2025). These approaches typically rely on manually engineered features, such as color histograms, texture descriptors, or shape features (Li et al., 2025b). By combining manual feature extraction with classifiers, they can distinguish between different disease types. To some extent, these methods can achieve satisfactory classification performance and adapt well to small-scale datasets. However, traditional machine learning still has notable limitations. First, manual feature extraction depends heavily on prior experience and struggles to capture the complex morphology and multi-scale characteristics of lesions. The recognition accuracy drops significantly when differences between diseases are subtle or when environmental interference is strong (Peng et al., 2024). Second, such methods cannot generally model spatial and contextual information in depth, making robust recognition challenging in complex field environments (Reek et al., 2024). Moreover, as the scale of agricultural imaging data continues to expand, traditional approaches show increasing limitations in feature representation and model generalization. This highlights the urgent need for more efficient and intelligent solutions.

In recent years, the rapid development of deep learning has created new opportunities for agricultural disease recognition (Fan et al., 2025; Wang et al., 2024b, 2022). Deep learning can automatically learn multi-level feature representations, eliminating the reliance on handcrafted features, and has demonstrated outstanding performance in tasks such as image classification and object detection (Zhang et al., 2025b, 2025e, 2026). For example, Pan et al. (2022) employed the YOLOX model to detect diseased regions in rice and integrated the detection results into a Siamese network for disease identification. Although this approach achieved promising results, its two-stage architecture led to higher computational complexity. To address this issue, Ahad et al. (2023) explored the effectiveness of classic end-to-end convolutional neural network (CNN) models for rice disease recognition, including DenseNet121, ResNet152, and ResNeXt101. Yang et al. (2023a) enhanced the GoogLeNet architecture with an attention mechanism, achieving higher recognition accuracy compared to networks such as AlexNet and ResNet34. Liu et al. (2024b) proposed the PSOC-DRCNet model to improve focus on diseased regions. However, most of these methods rely on complex and deep architectures with large numbers of parameters and significant computational costs, which restrict their deployment on embedded sensors and mobile devices. It is worth noting that some studies have also extended visual perception methods to paddy field scene structures and operational environments. For example, Chen et al. (2025) achieved end-to-end rice row detection through an instance segmentation framework, while Gong et al. (2024) improved positioning accuracy in indoor plant factory environments by integrating multisensor fusion, providing valuable references for intelligent perception applications in agriculture. Unlike these studies that mainly focus on scene structure modeling and navigation perception in paddy field environments, this work concentrates on the recognition of rice leaf disease images, with particular attention to fine-grained texture patterns and high- and low-frequency representations in the frequency domain. To this end, we design a lightweight wavelet-convolution-based high–low-frequency perception network to enhance the fine-grained discriminability of disease categories and improve the deployability of the model on resource-constrained platforms.

To meet the demand for high efficiency and low power consumption in practical applications, researchers have increasingly focused on the design and optimization of lightweight networks. For example, Zhang et al. (2025a) improved the MobileViT architecture to achieve accurate rice disease classification while maintaining a lightweight structure. Wang et al. (2025) based their model on EfficientNet and incorporated an attention mechanism, significantly reducing the number of parameters. Lv et al. (2025) proposed an enhanced lightweight ConvNeXt model that requires only 2.91 million parameters to accurately identify rice diseases. However, most existing lightweight models achieve efficiency primarily by compressing network depth and width. While this reduces parameter size and computational cost, it limits the ability to model fine-grained textures and frequency-domain features. High-frequency components often contain detailed textures and edge cues, which are crucial for identifying disease characteristics (Li et al., 2025a). Low-frequency components, on the other hand, represent overall structure and background semantics, which help enhance the robustness of the model. In agricultural disease recognition, lesions typically exhibit both coarse low-frequency shape variations and subtle high-frequency texture differences. Standard spatial-domain convolutions struggle to capture both types of information simultaneously. Currently, most lightweight models adjust attention distribution through depth reduction or attention modules while remaining within the spatial domain, overlooking the complementary nature of frequency-domain features. As a result, when identifying rice disease types with minute lesions or delicate texture patterns, these models often suffer a drop in accuracy.

To address the aforementioned issues, this work proposes a lightweight wavelet-enhanced high-low-frequency-aware network (LightWaveNet), which aims to effectively capture discriminative features of disease images across different frequency domains while maintaining a lightweight model, thus achieving more robust and efficient disease recognition. To fully leverage the complementarity of information from different frequency ranges, LightWaveNet incorporates a wavelet convolution-based high-low frequency decomposition mechanism into its backbone network. Through parallel wavelet convolution and pooling operations, the model efficiently constructs multi-frequency domain representations. Meanwhile, during downsampling, maximum pooling and average pooling are combined to balance the representation of fine lesion textures and global structural patterns. In addition, to alleviate gradient propagation difficulties and the insufficiency of single-stage supervision in deep networks, a multi-stage supervision learning strategy is designed. Auxiliary classification supervision is introduced at both shallow and mid-level feature extraction stages, which enhances the discriminative power of learned features and improves the generalization capability of the model. The main contributions of this work are as follows:

1. We propose a lightweight rice disease recognition network, named LightWaveNet. This network significantly reduces the number of parameters and computational complexity while maintaining recognition accuracy, making it suitable for deployment on resource-constrained mobile devices for real-time disease diagnosis.
2. A wavelet pooling module (WPM) is designed to capture the high- and low-frequency feature representations of rice disease images. This module employs a discrete wavelet transform to decompose the feature maps into multiple frequency sub-bands. This enables the network to explicitly and in parallel learn and model the low-frequency structural information and high-frequency texture details of diseases, thereby obtaining more discriminative feature representations.
3. We design a multi-stage supervision strategy by introducing auxiliary classifiers and loss functions in intermediate layers of the network. This strategy not only effectively alleviates the gradient vanishing problem during deep network training but also guides each layer to learn more discriminative features, thus improving the overall performance and convergence speed of the model.

The remainder of this work is organized as follows: Section 2 provides a detailed description of the dataset and the proposed LightWaveNet. Section 3 presents the experimental settings and results analysis. Section 4 discusses the results. Finally, Section 5 concludes the work.

## 2 Materials and methods

### 2.1 Data collection

**Figure 1:**
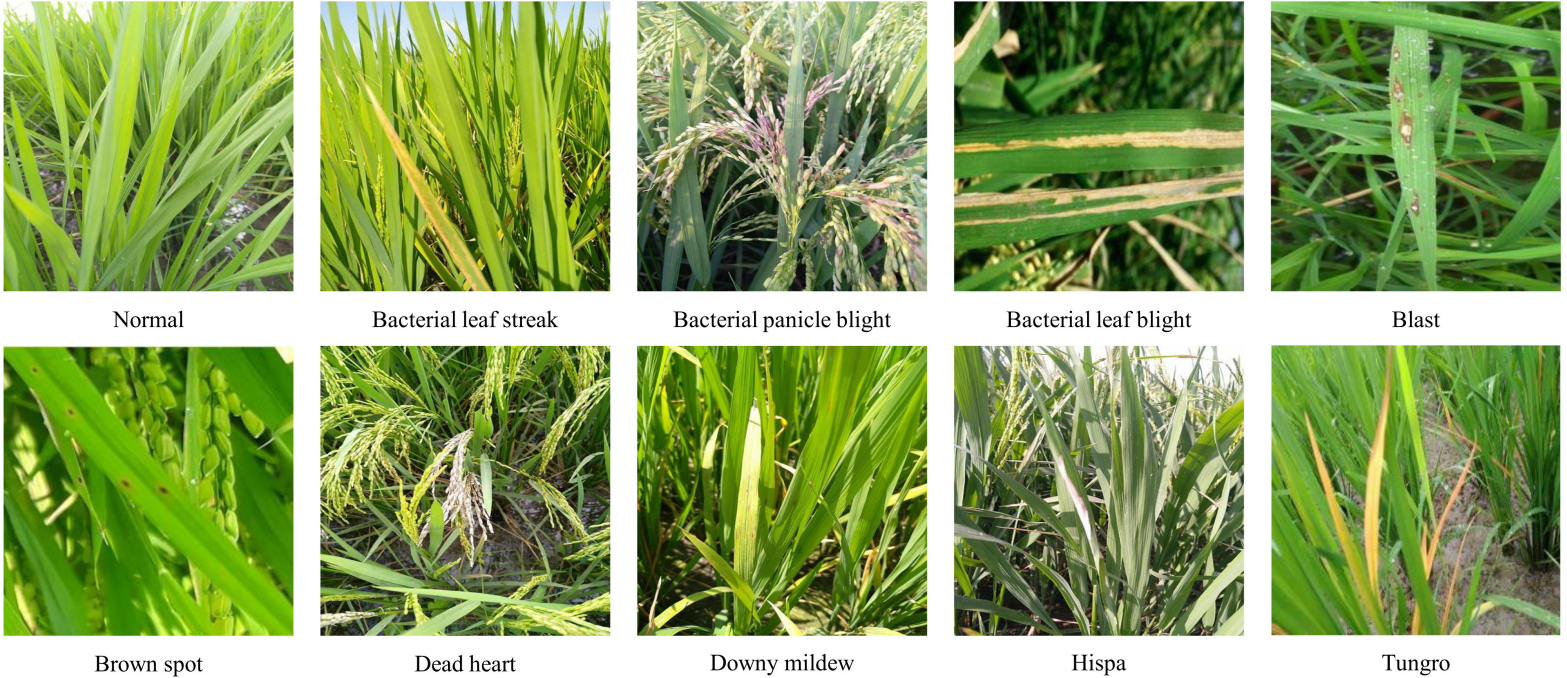
Example RGB images of rice diseases.

The dataset used in this study was compiled from both public datasets and online sources (e.g., Kaggle). The public dataset contains 5932 rice leaf disease images (Sethy et al., 2020), covering four classes: bacterial leaf blight, blast, brown spot, and tungro. In addition, to further enrich the sample diversity and disease categories, 7180 images were collected from the Kaggle platform and other agricultural disease image repositories. These images cover nine rice disease types as well as healthy rice leaves. The disease types include bacterial leaf streak, bacterial panicle blight, bacterial leaf blight, blast, brown spot, dead heart, downy mildew, hispa, and tungro. All collected images were manually screened and annotated to ensure data quality and accuracy. Finally, the two sources were merged into a single dataset containing 13,112 images in total. Examples of these disease images are shown in **Figure 1**.

### 2.2 Data processing

To match the model’s input size, all images were resized to a spatial resolution of 224×224 pixels using a bilinear interpolation algorithm. At the same time, color space normalization was applied to the images, scaling pixel values to the range of 0-1. To avoid the impact of class imbalance on model performance, data augmentation was performed on classes with fewer samples, including random rotation, horizontal and vertical flipping, as well as random brightness and contrast perturbations. After augmentation, the dataset contained 17,245 images. Finally, the dataset was randomly divided into training, validation, and test sets in a ratio of 5:1:4. The training set was used for model parameter learning, the validation set for hyperparameter tuning and overfitting prevention, and the test set for evaluating the final performance of the model. The detailed dataset partitioning is shown in **Table 1**.

**Table 1:** Sample division results of rice disease dataset.

No.	Class	Train	Validation	Test	Total
1	Normal	755	151	605	1511
2	Bacterial leaf streak	972	194	778	1944
3	Bacterial panicle blight	879	175	704	1758
4	Bacterial leaf blight	993	198	795	1986
5	Blast	1035	207	829	2071
6	Brown spot	988	197	791	1976
7	Dead heart	624	124	500	1248
8	Downy mildew	786	157	629	1572
9	Hispa	692	139	554	1385
10	Tungro	897	179	718	1794
Total	–	8621	1721	6903	17,245

### 2.3 Methodology

**Figure 2:**
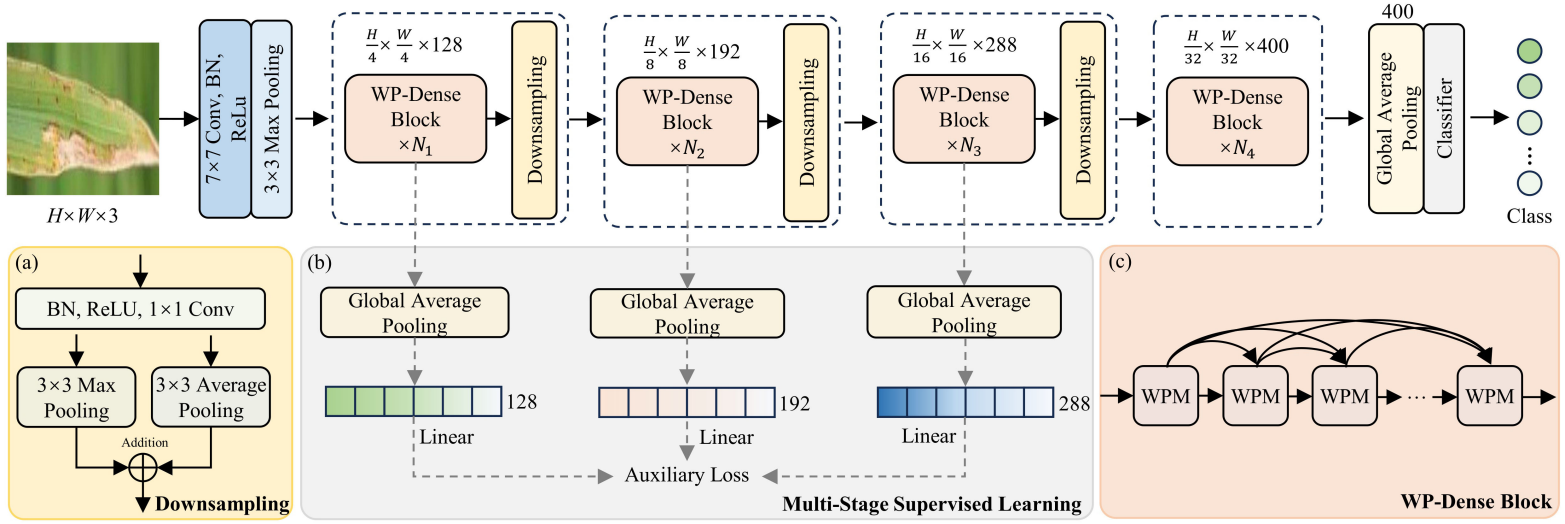
The proposed LightWaveNet network architecture. **(a)** Downsampling. **(b)** Multi-stage supervision structure. **(c)** Dense connections. The input size of the network is 224 × 224.

The schematic diagram of the proposed LightWaveNet architecture is shown in **Figure 2**. Similar to the learning paradigm of most image classification networks, LightWaveNet first employs a 7×7 convolution (Conv) and a 3×3 max pooling operation to extract shallow features from the image *X* ∈ ℝ*^H^*^×^*^W^*^×3^, where *H* and *W* denote the height and width of the image, respectively. It is worth noting that batch normalization (BN) and the ReLU activation function are applied to accelerate network convergence and enhance nonlinear representation. After shallow feature extraction, 64 feature maps are obtained. Subsequently, four stages of deep feature extraction modules are used to learn more discriminative semantic information, with each stage consisting of a wavelet pooling dense (WP-Dense) block followed by downsampling. Specifically, each WP-Dense block is composed of *N* WPM modules stacked in a densely connected manner. Compared with residual connections, dense connections enable more sufficient feature reuse and information flow between layers, thus alleviating the gradient vanishing problem and improving the ability to capture fine-grained features. In each dense connection, the number of WPMs is set to 2, 4, 6, and 8, respectively.

In the first three feature extraction stages, downsampling is used to reduce the spatial resolution of the image and compress the number of feature maps. As a result, the spatial resolutions of the feature maps generated in each stage are *H/*4 × *W/*4, *H/*8 × *W/*8, *H/*16 × *W/*16, and *H/*32 × *W/*32, with feature dimensions of 128, 192, 288, and 400, respectively. In addition, LightWaveNet incorporates multi-stage supervised learning in the first three stages. Specifically, at the end of the dense block in each of the first three stages, global average pooling and a linear classifier are applied to compute auxiliary losses. This mechanism effectively constrains shallow and intermediate features during network training, thereby enhancing the model’s feature representation capability. It is worth noting that multi-stage supervised learning is only used during training. After the four stages of feature extraction, global average pooling is applied to aggregate spatial information, resulting in a 400-dimensional feature vector, which is then passed through a linear classifier to output the final rice disease class. The network implementation code is available at the following link: https://github.com/zhang2508/LightWaveNet.

#### 2.3.1 Wavelet convolution

To simultaneously capture multi-scale spatial structures and multi-frequency information, we introduce a convolutional module based on 2D discrete wavelet transform (WT) in the feature extraction stage, named wavelet transform convolution (WTConv). The detailed principles of WTConv can be referred to in the work of Finder et al. (2025). Specifically, a single-layer 2D Haar wavelet transform is first applied to decompose the input feature map *X* into a low-frequency approximation component *X_LL_* and high-frequency detail components in the horizontal, vertical, and diagonal directions, denoted as *X_LH_*, *X_HL_*, and *X_HH_*, respectively. This process can be expressed as Equation 1:

(1) $$\left[X_{LL},X_{LH},X,X_{HH}\right]=\text{WT}\left(X\right)$$

where *WT*(·) is implemented using a set of 2 × 2 orthogonal filters. Each sub-band is downsampled by half spatial resolution and processed by a depthwise separable convolution to extract frequency-domain features, as formulated below in Equation 2:

(2) $$Y_{s}=W_{\left(k\times k\right)}*\;X_{s}+b_{s},\;s\in \left\{LL,LH,HL,HH\right\}$$

where ∗ denotes the 2D convolution operation, *W*_(_*_k_*_×_*_k_*_)_ represents the convolution kernel of size *k* × *k*, and *b_s_* is the bias term.

To further enlarge the receptive field, the low-frequency component *X_LL_* can be recursively decomposed for *l* levels (i.e., multi-level WT). The decomposition at the *i*-th level is given by Equation 3:

(3) $$\left[X_{LL}^{\left(i\right)},X_{LH}^{\left(i\right)},X_{HL}^{\left(i\right)},X_{HH}^{\left(i\right)}\right]=\text{WT}\left(X_{LL}^{\left(i-1\right)}\right)$$

where $X_{LL}^{\left(0\right)}=X$. After the convolution, the results from each frequency band are reconstructed back to the original spatial domain using the inverse wavelet transform (IWT), and the final output is obtained via channel-wise weighted fusion. This process can be expressed by Equation 4:

(4) $$Y=\text{IWT}\left({\left\{Y_{LL}^{\left(i\right)},Y_{LH}^{\left(i\right)},Y_{HL}^{\left(i\right)},Y_{HH}^{\left(i\right)}\right\}}_{i=1}^{l}\right)$$

**Figure 3:**
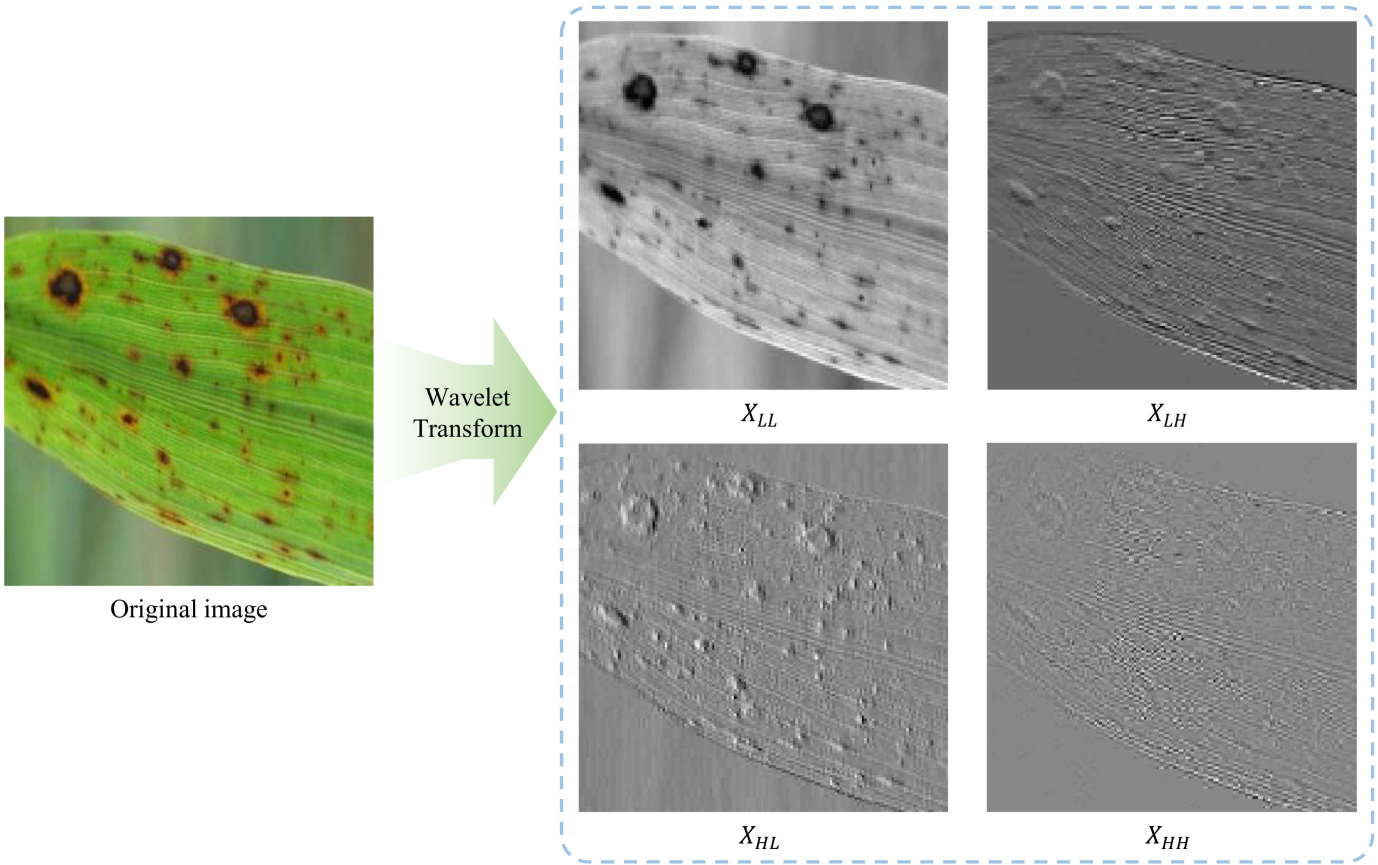
Illustration of 2D Haar wavelet transform decomposition.

**Figure 3** illustrates the 2D Haar wavelet decomposition of a rice leaf image affected by disease. The original image is decomposed into four sub-bands: *X_LL_*, *X_LH_*, *X_HL_*, and *X_HH_*. Among these, *X_LL_* preserves the overall structural and low-frequency information, whereas *X_LH_*, *X_HL_*, and *X_HH_* capture complementary high-frequency components such as edges, textures, and fine lesion boundaries. From the figure, the edges and textures of rice leaf lesions are significantly highlighted and enhanced in the *X_LH_* and *X_HL_* components. At the same time, *X_LL_* retains the macroscopic structure and energy distribution of the image, namely the overall morphological information of the leaf and the spatial context of the lesions. This decomposition effectively separates the lesions from the normal leaf texture, allowing the model to learn complementary representations from both high- and low-frequency domains. By integrating these multi-frequency cues, the accuracy of rice disease recognition can be effectively enhanced.

#### 2.3.2 Dense connections

To enhance feature propagation and gradient flow, a dense connection mechanism (Huang et al., 2017) is introduced into the feature extraction stage of the network, as shown in **Figure 2c**. In this mechanism, the input to each layer is obtained by concatenating the outputs of all preceding layers along the channel dimension. Let the network consist of *N* sequentially stacked convolutional layers, and denote the input feature map of the *i*-th layer as $x_{i}$; then, the output of this layer can be formulated as Equation 5:

(5) $$x_{i}=H_{i}\left(\left[x_{0},x_{1},\ldots ,x_{i-1}\right]\right)$$

where *x*_0_ is the initial input to the network, [·] denotes channel-wise concatenation, and *H_i_*(·) represents the nonlinear transformation function of the *i*-th layer (i.e., WPM).

If each layer generates *k* feature maps (i.e., the growth rate), the number of input channels for the *i*-th layer is given by Equation 6:

(6) $$C_{i}=C_{0}+k\times \left(i-1\right)$$

where $C_{0}$ is the number of initial input channels to the dense block. Accordingly, the number of output channels for the *i*-th layer can be expressed as Equation 7:

(7) $$C_{out}=C_{0}+k\times i$$

#### 2.3.3 Wavelet pooling module

**Figure 4:**
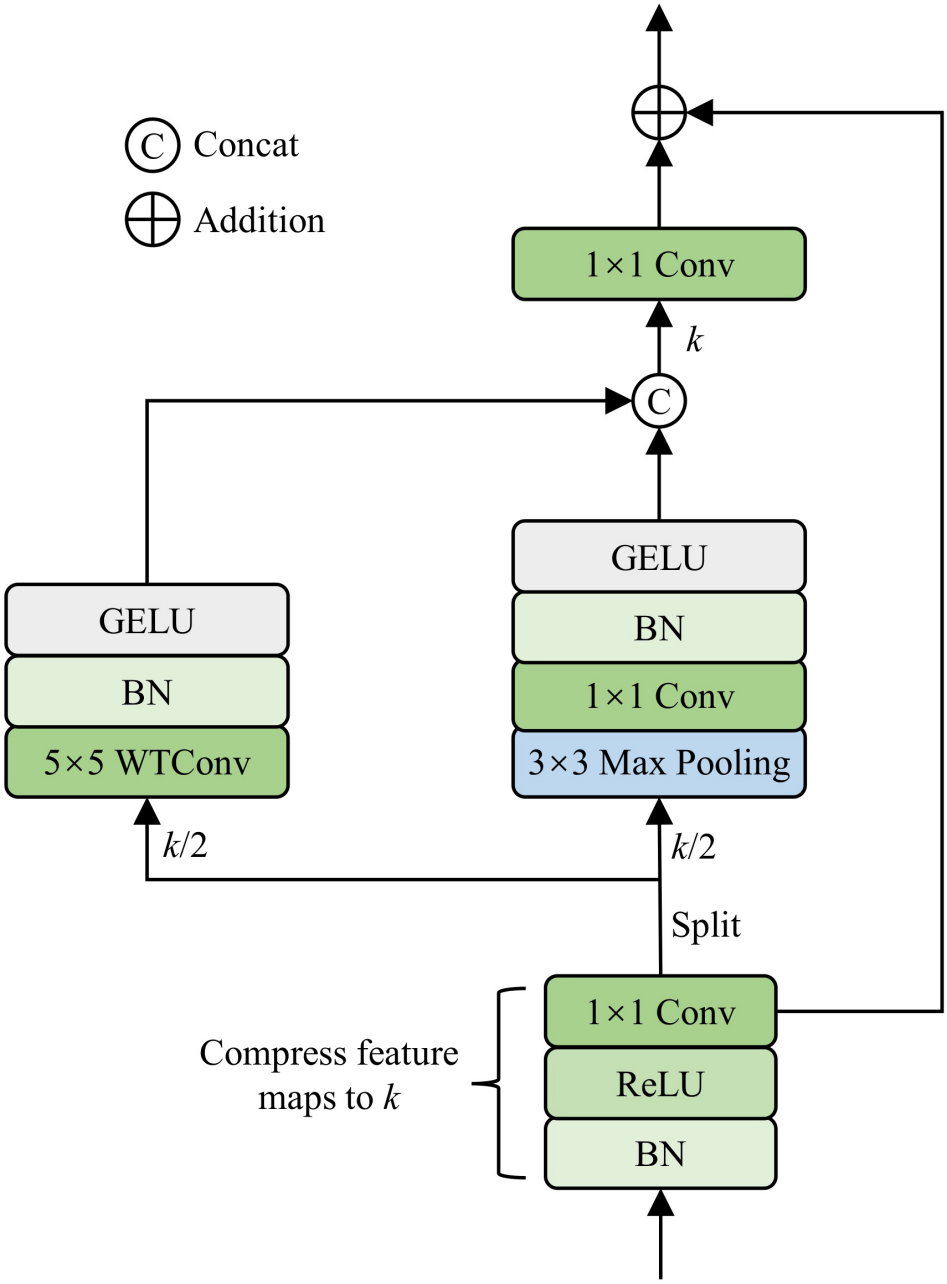
Structure of the WPM module.

In rice disease identification, lesions and streaks on the surface of leaves often contain both low-frequency global distribution information and high-frequency fine-grained textures. Therefore, the WPM module is designed as the feature extraction structure within the dense connections, aimed at capturing multi-scale high- and low-frequency feature variations in rice disease images. The structure of WPM is shown in **Figure 4**. Specifically, WPM receives the output from the previous dense block and applies a compression function to reduce the feature dimension to *k*, thereby alleviating the computational burden of the network. This compression function consists of BN, a ReLU activation function, and a 1×1 convolution, as formulated below in Equation 8:

(8) $$Z=\text{Con}{\text{v}}_{\left(1\times 1\right)}\left(\sigma \left(\text{BN}\left(X\right)\right)\right)$$

where $\text{Con}{\text{v}}_{\left(1\times 1\right)}\left(\cdot \right)$ denotes a convolution with a 1×1 kernel, *σ*(·) denotes the ReLU activation function, and BN(·) denotes the batch normalization layer. Next, the compressed feature map is equally split along the channel dimension into two sub-feature maps, *X*_1_ and *X*_2_, each with *k/*2 channels. Then, *X*_1_ and *X*_2_ are processed by two parallel branches, respectively. Specifically, the first branch employs a 5×5 wavelet convolution to capture the low-frequency global contextual information of rice leaf lesions, which can be expressed as Equation 9:

(9) $$Y_{1}=\text{GELU}\left(\text{BN}\left({\text{WTConv}}_{\left(5\times 5\right)}\left(X_{1}\right)\right)\right)$$

where GELU(·) denotes the GELU activation function, and ${\text{WTConv}}_{\left(5\times 5\right)}\left(\cdot \right)$ denotes the 5×5 wavelet convolution. The second branch utilizes 3×3 max pooling to capture high-frequency fine-grained texture features of the disease, as formulated in Equation 10:

(10) $$Y_{2}=\text{GELU}\left(\text{BN}\left({\text{Conv}}_{\left(1\times 1\right)}\left({\text{MaxPool}}_{\left(3\times 3\right)}\left(X_{2}\right)\right)\right)\right)$$

where ${\text{MaxPool}}_{\left(3\times 3\right)}\left(\cdot \right)$ denotes the max pooling operation with a 3×3 kernel. The outputs *Y*_1_ and *Y*_2_ from the two branches are concatenated along the channel dimension and fused through a 1×1 convolution. Finally, a residual connection is applied to enhance information flow within the network. This can be formulated as Equation 11:

(11) $$Z={\text{Conv}}_{\left(1\times 1\right)}\left(\text{Concat}\left(Y_{1},Y_{2}\right)\right)+X$$

where Concat(·) denotes concatenation along the channel dimension.

#### 2.3.4 Downsampling

The downsampling structure is shown in **Figure 2a**. This module adopts a dual-branch pooling architecture, which can effectively preserve both high- and low-frequency details of the image while reducing computational resource consumption. Specifically, the downsampling module receives the output from the dense block, first normalizes the features through a BN layer, and then applies a ReLU activation function to enhance nonlinear representation. Subsequently, a 1×1 convolution is used to reduce the number of channels in the feature map. The number of filters in the convolution kernel is controlled by a compression coefficient *θ*, set to 0.5. This means that after downsampling, the number of feature maps is reduced by half. This process can be expressed as Equation 12:

(12) $$F^{\prime }={\text{Conv}}_{\left(1\times 1\right)}\left(\sigma \left(\text{BN}\left(F_{\text{in}}\right)\right)\right)$$

Next, the feature map $F^{\prime }$ is fed into two parallel pooling branches. The first branch adopts 3×3 max pooling to enhance high-frequency features such as lesion edges and fine-grained textures. The other branch adopts 3×3 average pooling to retain low-frequency information such as the overall leaf shape and background distribution. Finally, the outputs of the two branches are fused through element-wise addition to achieve complementary representation of high- and low-frequency information, completing the downsampling process. This can be defined as Equation 13:

(13) $$F_{\text{out}}={\text{MaxPool}}_{\left(3\times 3\right)}\left(F^{\prime }\right)+{\text{AvgPool}}_{\left(3\times 3\right)}\left(F^{\prime }\right)$$

where ${\text{AvgPool}}_{\left(3\times 3\right)}\left(\cdot \right)$ denotes the average pooling operation with a 3×3 kernel.

#### 2.3.5 Multi-stage supervised learning

In deep network training, if supervision signals are only provided at the final layer, intermediate layers may lack sufficient feature constraints, making it difficult to learn effective feature representations, which in turn affects the classification performance of the network. To enhance the discriminative power of shallow features, this study introduces a multi-stage supervision optimization mechanism into the classification network. Specifically, auxiliary loss branches are added to the dense connection blocks in the first three stages to perform supervised learning on the intermediate layer features, thereby improving the multi-level feature representation capability of the network. For the feature map *F*^(^*^i^*^)^ output by the *i*-th stage (*i* ∈ {1,2,3}), we aggregate it into a feature vector *v*^(^*^i^*^)^ via global average pooling (GAP). As such, the first three stages will yield feature vectors of sizes 128, 192, and 288, respectively. This process can be expressed as Equation 14:

(14) $${ʋ}^{\left(i\right)}=\text{GAP}\left(F^{\left(i\right)}\right)$$

where GAP(·) denotes the global average pooling function. Next, the feature vector at each stage is passed through a linear projection function to compute the class prediction probabilities, as shown below in Equation 15:

(15) $${\hat{y}}^{\left(i\right)}=\text{Softmax}\left(W^{\left(i\right)}x^{\left(i\right)}+b^{\left(i\right)}\right)$$

where Softmax(·) denotes the Softmax activation function, and $W^{\left(i\right)}$ and $b^{\left(i\right)}$ are the weight and bias for the *i*-th stage, respectively. Subsequently, the auxiliary loss for different stages is calculated using the cross-entropy loss function, as expressed by Equation 16:

(16) $$L_{\text{aux},i}=-\sum_{c=1}^{C}y_{c}^{\left(i\right)}\text{log }\left({\hat{y}}_{c}^{\left(i\right)}\right)$$

where $L_{\text{aux},i}$ denotes the auxiliary loss of the *i*-th stage, and $y_{c}^{\left(i\right)}$ and ${\hat{y}}_{c}^{\left(i\right)}$ denote the one-hot label vector and class prediction probability for the *i*-th stage, respectively. Finally, the total network loss consists of the main loss *L*_main_ from the backbone network and the auxiliary losses *L*_aux_. This can be expressed as Equation 17:

(17) $$L_{\text{total}}=\left(1-\lambda \right)L_{\text{main}}+\lambda \sum_{i=1}^{3}L_{\text{aux},i}$$

where *λ* is the weight coefficient for the loss function, which is set to 0.1 in this work.

### 2.4 Evaluation metrics

In this study, precision, recall, F1-score, and accuracy are selected as evaluation metrics to assess the recognition performance of the model. Among them, precision represents the proportion of samples predicted as positive that are actually positive, and recall represents the proportion of actual positive samples that are correctly predicted. F1-score is the harmonic mean of precision and recall, used to balance precision and recall. Accuracy represents the proportion of correctly classified samples among all predictions. Their calculation formulas are as follows in Equations 18–21:

(18) $$\text{Precision}=\frac{\text{TP}}{\text{TP}+\text{FP}}$$

(19) $$\text{Recall}=\frac{\text{TP}}{\text{TP}+\text{FN}}$$

(20) $$\text{F}1-\text{score}=\frac{2\times \text{Precision}\times \text{Recall}}{\text{Precision}+\text{Recall}}$$

(21) $$\text{Accuracy}=\frac{\text{TP}+\text{TN}}{\text{TP}+\text{TN}+\text{FP}+\text{FN}}$$

where true positive (TP) denotes the number of samples predicted as positive and actually positive, false positive (FP) denotes the number of samples predicted as positive but actually negative, false negative (FN) denotes the number of samples predicted as negative but actually positive, and true negative (TN) denotes the number of samples predicted as negative and actually negative. In addition, the number of parameters and floating-point operations (FLOPs) are chosen to measure the computational complexity of the model. Parameters reflect the storage requirements of the network, while FLOPs represent the amount of computation required for a single forward propagation of the model.

## 3 Results

### 3.1 Experimental setup

The experiments in this study were conducted in a Linux system environment. The hardware configuration includes a 24 GB NVIDIA GeForce RTX 3090 graphics processing unit, an Intel(R) Xeon(R) Gold 6148 processor, and 64 GB of memory. The programming language used was Python 3.10, and the deep learning framework adopted was PyTorch 2.3.0. During model training, the batch size was set to 32, the learning rate was 0.001, the number of training epochs was 100, the optimizer chosen was Adam, and the loss function was the cross-entropy loss function. It should be emphasized that this study did not employ a learning rate scheduling strategy, weight decay, or an early stopping mechanism. In addition, five-fold cross-validation was employed for training and evaluating the dataset.

### 3.2 Ablation experiment

To verify the effectiveness of each module in LightWaveNet, ablation experiments were conducted, and the results are shown in **Table 2**. The bold values in the table indicate the best results, similarly hereinafter. As can be seen from the results in the table, all the proposed modules play a positive role in improving the model performance. When using only WTConv (method a), the precision, recall, F1-score, and accuracy were 93.92%, 93.88%, 93.86%, and 93.90%, respectively. On this basis, introducing the WPM (method b) led to significant improvements in all four metrics, with recall increasing by 0.83 percentage points, indicating enhanced detection capability for targets. Further incorporating the downsampling module (method c) resulted in continued improvements in precision and accuracy, suggesting that this module helps reduce the false detection rate while maintaining recall. Finally, based on method c, the introduction of multi-stage supervised learning (method d) allowed the model to achieve the highest performance across all metrics, with precision, recall, F1-score, and accuracy reaching 95.72%, 95.89%, 95.80%, and 95.90%, respectively, representing an overall improvement of approximately 1.8-2.0 percentage points compared to the baseline method a. This demonstrates that multi-stage supervision can more effectively optimize feature representation, thereby significantly enhancing the overall recognition capability of the model. Furthermore, taking Method b as the baseline, we conducted a significance analysis using McNemar’s test with a significance level of 0.05. The results show that the p-values obtained after adding each module are all lower than the significance threshold (p< 0.05), indicating that the performance improvements brought by the added modules are statistically significant. In summary, after integrating all modules, LightWaveNet achieves the highest recognition accuracy.

**Table 2:** Ablation experiment results.

Method	WTConv	WPM	Downsampling	Multi-stage supervised learning	Precision (%)	Recall (%)	F1-score (%)	Accuracy (%)	P-value
a	✓				93.92	93.88	93.86	93.9	–
b	✓	✓			94.47	94.71	94.57	94.71	0.0016
c	✓	✓	✓		94.8	94.62	94.69	94.77	0.0018
d	✓	✓	✓	✓	**95.72**	**95.89**	**95.80**	**95.90**	0.0001

Note: Bold values indicate the best results, and “√” indicates that the module is used.

**Figure 5:**
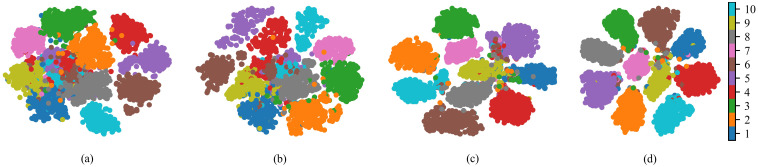
t-SNE feature visualizations of different methods. **(a)** Method a. **(b)** Method b. **(c)** Method c. **(d)** Method d.

**Figure 5** presents the t-SNE feature visualization results for different methods. The visualization results intuitively reflect the trends in inter-class separability and intra-class compactness in the feature space under each method. For method a, there is significant overlap in the distributions of samples from different classes, with blurred inter-class boundaries. In comparison, after adding the WPM, the boundaries of some categories become clearer, although the clustering of samples within the same class remains relatively dispersed. With the further introduction of the Downsampling module, the spacing between clusters of different classes increases significantly, enhancing inter-class separability while making the intra-class sample distribution more compact. This indicates that the module helps preserve both high- and low-frequency information of the image during the downsampling process. Finally, when multi-stage supervised learning is added on top of method c, the results of method d exhibit optimal separability: different categories form distinct and well-separated cluster structures with almost no apparent overlap, and intra-class consistency is markedly improved. This further demonstrates the effectiveness of multi-stage supervised learning. These findings are consistent with the quantitative results in the table, indicating that the collaborative contribution of all modules effectively enhances the discriminative power of the features and the recognition performance of the model.

**Figure 6:**
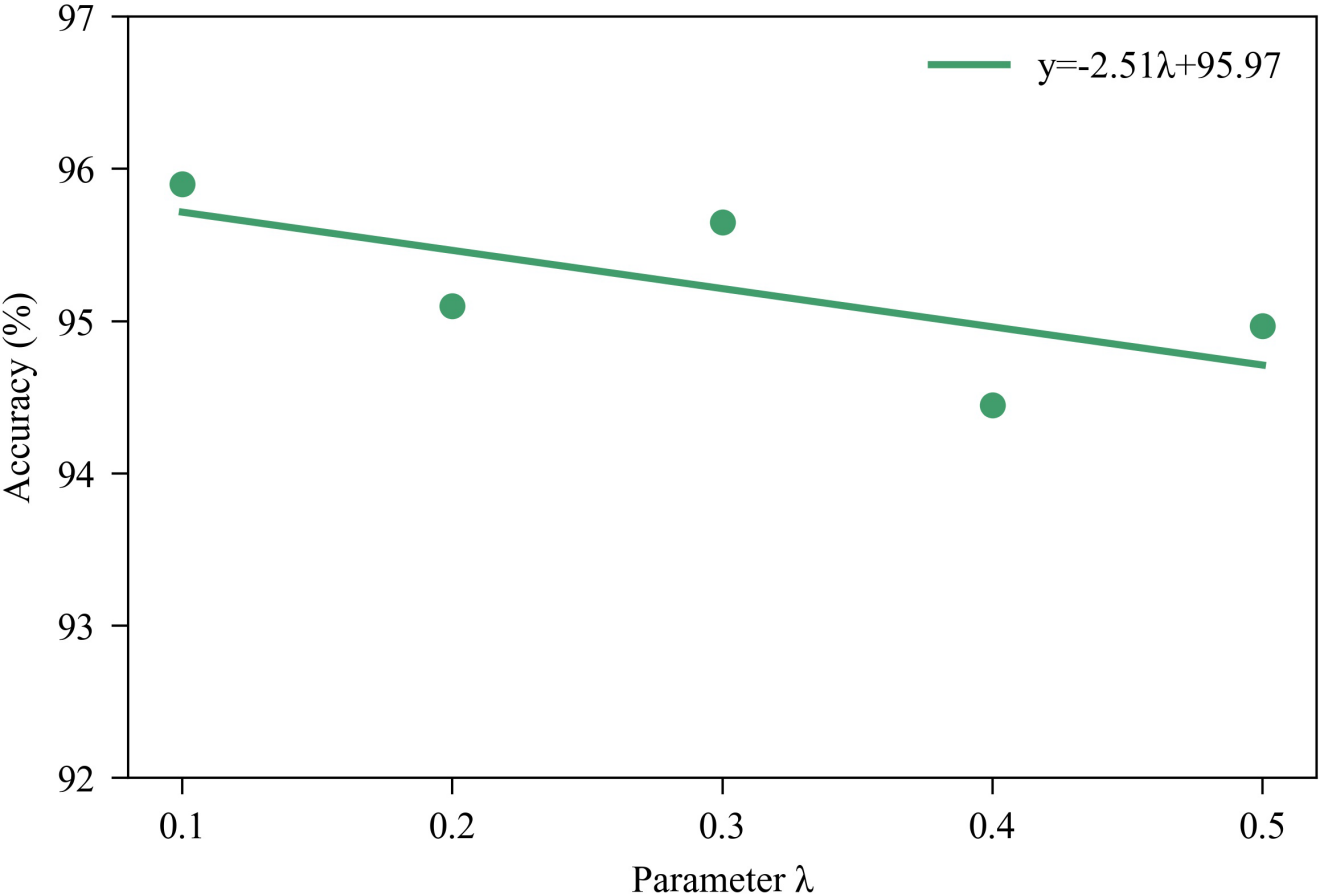
Results for different *λ* parameters.

To investigate the impact of the *λ* parameter in multi-stage supervised learning on model performance, we analyzed it with different *λ* values, as shown in **Figure 6**. Theoretically, applying supervisory constraints to each stage of the network helps introduce more discriminative semantic information into shallow features. As illustrated in the figure, the overall accuracy of the model shows a downward trend as *λ* increases. This may be because an excessively large *λ* causes the loss from the early stages to become overly dominant in the overall optimization, leading the model to overfit shallow features and thereby weakening the representational capacity of deeper features. In contrast, a smaller *λ* can provide the necessary supervision for shallow layers while preserving more optimization space for deep feature learning, thus achieving a better performance balance. The feature visualization results in **Figure 5** corroborate this observation. In summary, applying multi-stage supervision constraints to the network helps make full use of feature representations at different levels, thereby improving overall recognition accuracy.

### 3.3 Comparison with other algorithms

To evaluate the recognition performance of LightWaveNet, this study compares it with 15 state-of-the-art methods, including 9 heavyweight models and 6 lightweight models. The heavyweight models include ConvNeXt (Liu et al., 2022), DenseNet201 (Huang et al., 2017), EfficientNet (Tan and Le, 2019), GFNet (Rao et al., 2023), RepViT (Wang et al., 2024a), ResNet101 (He et al., 2015), ViM (Zhu et al., 2024), ViT (Dosovitskiy et al., 2021), and ResMamba (Zhang et al., 2025d). The lightweight models include DeiT-tiny (Touvron et al., 2021), GhostNet (Han et al., 2020), MobileNetV2 (Sandler et al., 2018), MobileNetV3 (Howard et al., 2019), MobileViT (Mehta and Rastegari, 2022), and ShuffleNet (Zhang et al., 2018). All models were configured according to their original papers and run under the same environment. The comparison results are shown in **Table 3**.

**Table 3:** Comparison results with other methods.

Model	Precision (%)	Recall (%)	F1-score (%)	Accuracy (%)
ConvNeXt	88.36	88.62	88.44	88.69
Densenet201	94.44	94.59	94.46	94.45
EfficientNet	92.53	92.74	92.59	92.71
GFNet	74.02	73.87	73.73	74.68
RepViT	83.82	84.10	83.81	84.17
ResNet101	90.64	90.87	90.66	90.70
ViM	73.19	73.25	73.10	73.92
ViT	62.97	62.18	62.25	63.49
ResMamba	86.85	86.85	86.68	87.06
DeiT-tiny	74.05	73.31	73.50	74.39
GhostNet	88.65	88.78	88.62	88.90
Mobilenetv2	91.37	91.69	91.46	91.63
Mobilenetv3	92.57	92.92	92.66	92.76
MobileViT	89.59	89.72	89.60	89.76
ShuffleNet	89.83	89.43	89.57	89.69
LightWaveNet	95.72	95.89	95.80	95.90

From the table, it can be seen that LightWaveNet significantly outperforms all other comparison methods across all evaluation metrics, with precision, recall, F1-score, and accuracy reaching 95.72%, 95.89%, 95.80%, and 95.90%, respectively. Compared with heavyweight models, LightWaveNet surpasses the second-ranked DenseNet201, with improvements of 1.28%, 1.30%, 1.34%, and 1.45% in precision, recall, F1-score, and accuracy, respectively, indicating that LightWaveNet has stronger feature extraction and discrimination capabilities. For some heavyweight methods (such as ViT, ViM, and GFNet), LightWaveNet achieves substantial performance boosts, with F1-score improvements exceeding 20 percentage points. Compared with lightweight models, LightWaveNet also leads significantly in both precision and recall. For example, compared with MobileNetV3, which ranks second among lightweight models, LightWaveNet improves precision and recall by 3.15% and 2.97%, respectively, while maintaining the advantage of a lightweight design. This demonstrates that LightWaveNet has significant advantages in balancing high accuracy and high efficiency, and can provide more accurate recognition results under resource-constrained conditions in practical applications.

**Figure 7:**
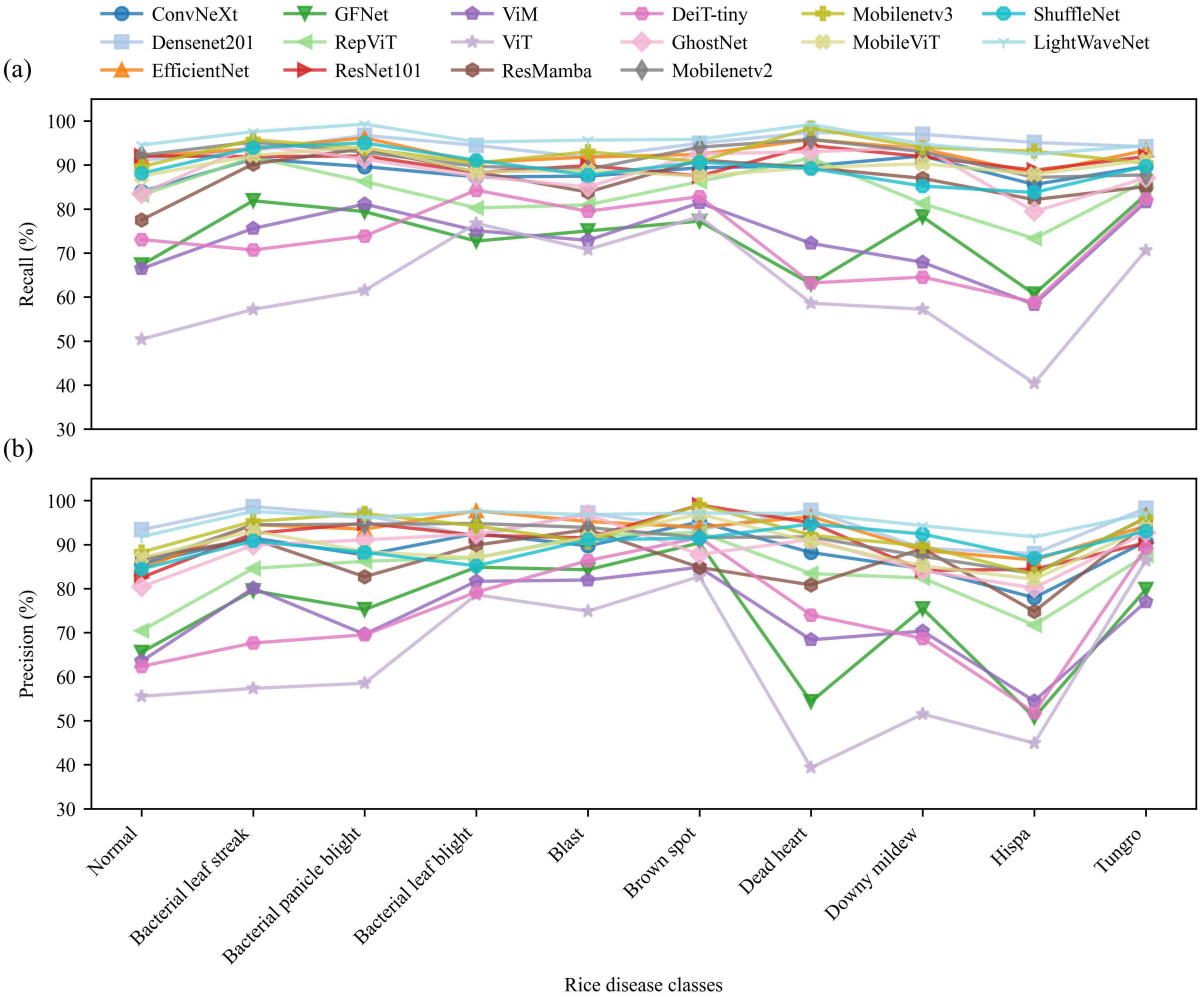
Precision and recall results for each class. **(a)** Precision for each class. **(b)** Recall for each class.

**Figure 7** further presents the comparative results of Precision and Recall for each class. As shown in the figure, most mainstream models maintain relatively high performance across the majority of classes, yet noticeable fluctuations still occur in a few complex ones. Overall, the curves of LightWaveNet are comparatively smoother with smaller oscillation amplitudes across classes, indicating better stability in rice disease recognition. In particular, for classes such as Normal, Blast, Brown spot, and Dead heart, both Recall and Precision remain at the higher end among all compared methods. In contrast, lightweight or pure vision Transformer models such as ViT, GhostNet, and DeiT-tiny show obvious performance drops in some classes (e.g., Hispa, Downy mildew, Tungro), reflecting an unstable trend. Traditional CNN models (e.g., ResNet101, EfficientNet) exhibit generally stable performance, but inconsistencies between Precision and Recall still occur in certain classes. In summary, LightWaveNet achieves a good balance between high Precision and Recall, with relatively consistent recognition performance across all classes, demonstrating stronger robustness and generalization ability when facing morphological differences among various rice diseases.

**Figure 8:**
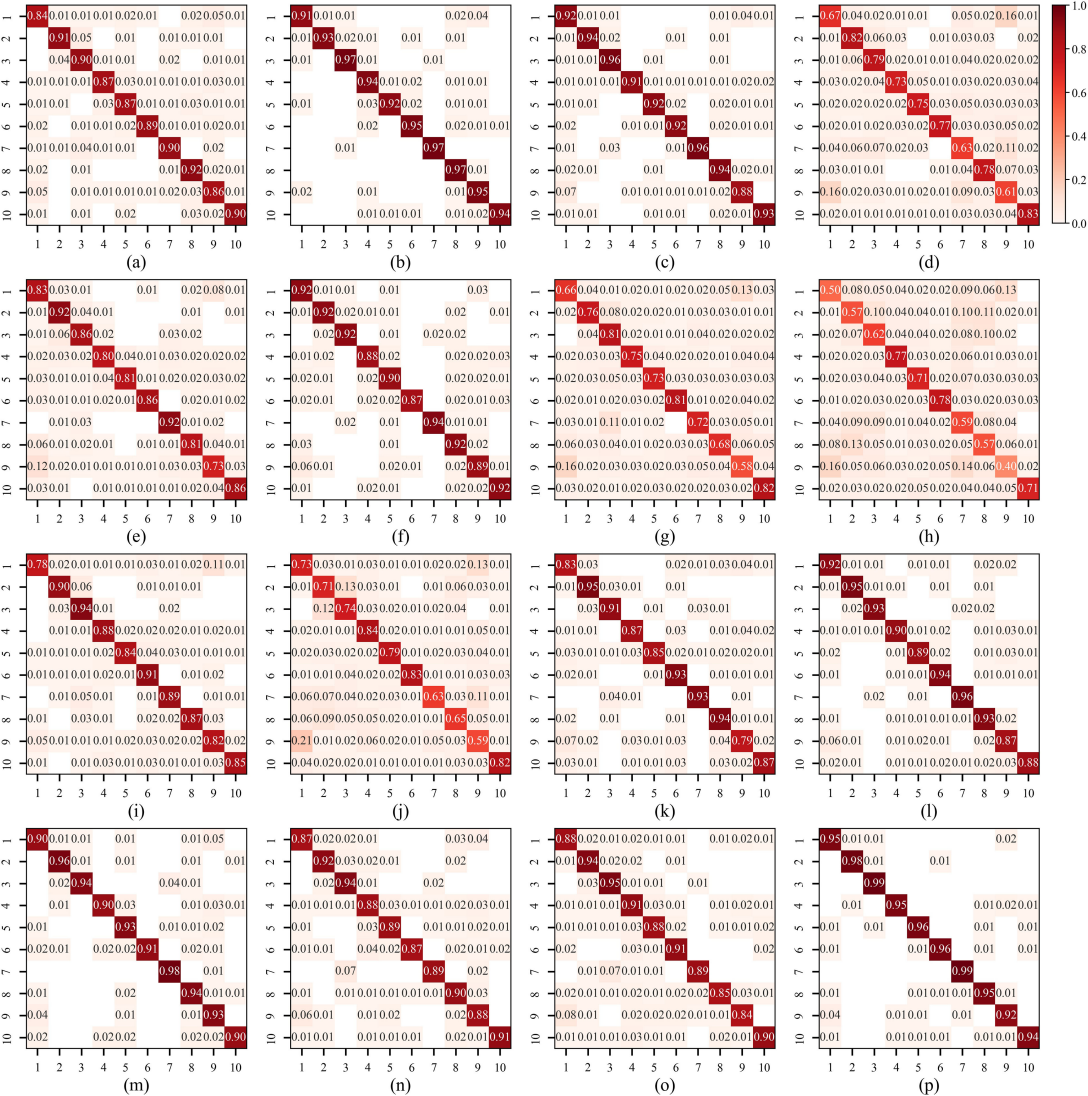
Confusion matrix results of the compared methods. **(a)** ConvNeXt. **(b)** DenseNet201. **(c)** EfficientNet. **(d)** GFNet. **(e)** RepViT. **(f)** ResNet101. **(g)** ViM. **(h)** ViT. **(i)** ResMamba. **(j)** DeiT-tiny. **(k)** GhostNet. **(l)** MobileNetV2. **(m)** MobileNetV3. **(n)** MobileViT. **(o)** ShuffleNet. **(p)** LightWaveNet.

From the confusion matrix results in **Figure 8**, it can be observed that there are significant differences among methods in terms of classification accuracy and the degree of confusion between categories. Many comparison methods, such as GFNet, ViT, and ViM, exhibit relatively high misclassification rates in certain categories. Specifically, these methods show noticeable cross-predictions between multiple classes, reflected by the presence of numerous dark-colored blocks in non-diagonal positions, indicating insufficient feature discrimination capability. Even for better-performing heavyweight models, such as DenseNet201, there is still some confusion between similar categories, leading to a drop in recall for certain classes. For lightweight models, such as DeiT-tiny and ShuffleNet, there is an evident distributional spread in predictions across multiple categories, resulting in higher error rates. In contrast, the proposed LightWaveNet’s confusion matrix displays a near-ideal diagonal distribution, with extremely low values for non-diagonal elements, almost eliminating significant inter-class confusion. This indicates that the method achieves stable and highly separable feature representations across all categories. These results are consistent with the quantitative evaluation metrics in **Table 3** and further validate LightWaveNet’s advantages in improving classification accuracy, reducing misclassification, and enhancing generalization ability.

### 3.4 Feature visualization

**Figure 9:**
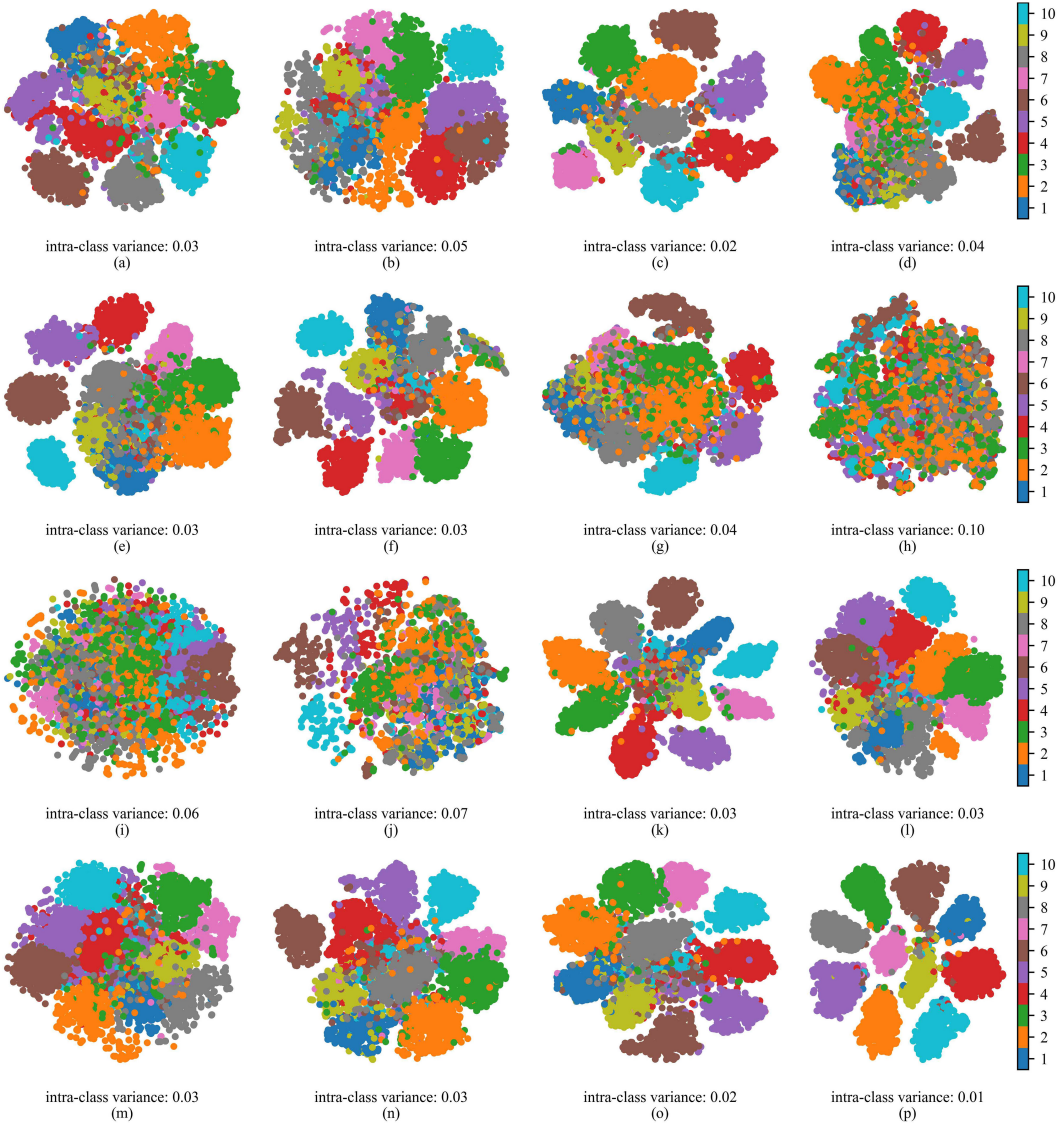
t-SNE feature visualizations of each model. **(a)** ConvNeXt. **(b)** DenseNet201. **(c)** EfficientNet. **(d)** GFNet. **(e)** RepViT. **(f)** ResNet101. **(g)** ViM. **(h)** ViT. **(i)** ResMamba. **(j)** DeiT-tiny. **(k)** GhostNet. **(l)** MobileNetV2. **(m)** MobileNetV3. **(n)** MobileViT. **(o)** ShuffleNet. **(p)** LightWaveNet.

To further analyze the differences in feature extraction among different models, a t-SNE feature visualization analysis was conducted, with the results shown in **Figure 9**. From the visualizations, it can be observed that different models exhibit significant differences in inter-class separability and intra-class compactness, with most comparison methods showing clear feature distribution overlaps. Specifically, models such as ViT, ResMamba, and DeiT-tiny have very blurred class boundaries, with many samples from different classes mixed, indicating insufficient discriminative capability in feature extraction. Some models, including ConvNeXt, ResNet101, and MobileNetV2, can separate certain categories, but still present a considerable number of overlapping regions. Such overlaps can cause the classifier to experience confusion during decision-making. In contrast, LightWaveNet’s feature distribution is much clearer: different classes form distinct and compact clusters with large inter-class distances and almost no intersections. This demonstrates that LightWaveNet can more effectively enhance inter-class differences while maintaining intra-class consistency during the feature extraction stage. Such a feature distribution helps the model make stable and accurate predictions, which is consistent with the quantitative analysis results and validates LightWaveNet’s outstanding feature representation capability and generalization performance. In addition, to quantitatively analyze the t-SNE visualization results, we calculated the intra-class variance of the projected feature embeddings. A lower variance indicates that samples within the same class are more compact. The results show that, among all compared models, the proposed model achieves the lowest average intra-class variance, which is consistent with the more compact clustering patterns observed in the visualization.

### 3.5 Visual display of recognition results

**Figure 10:**
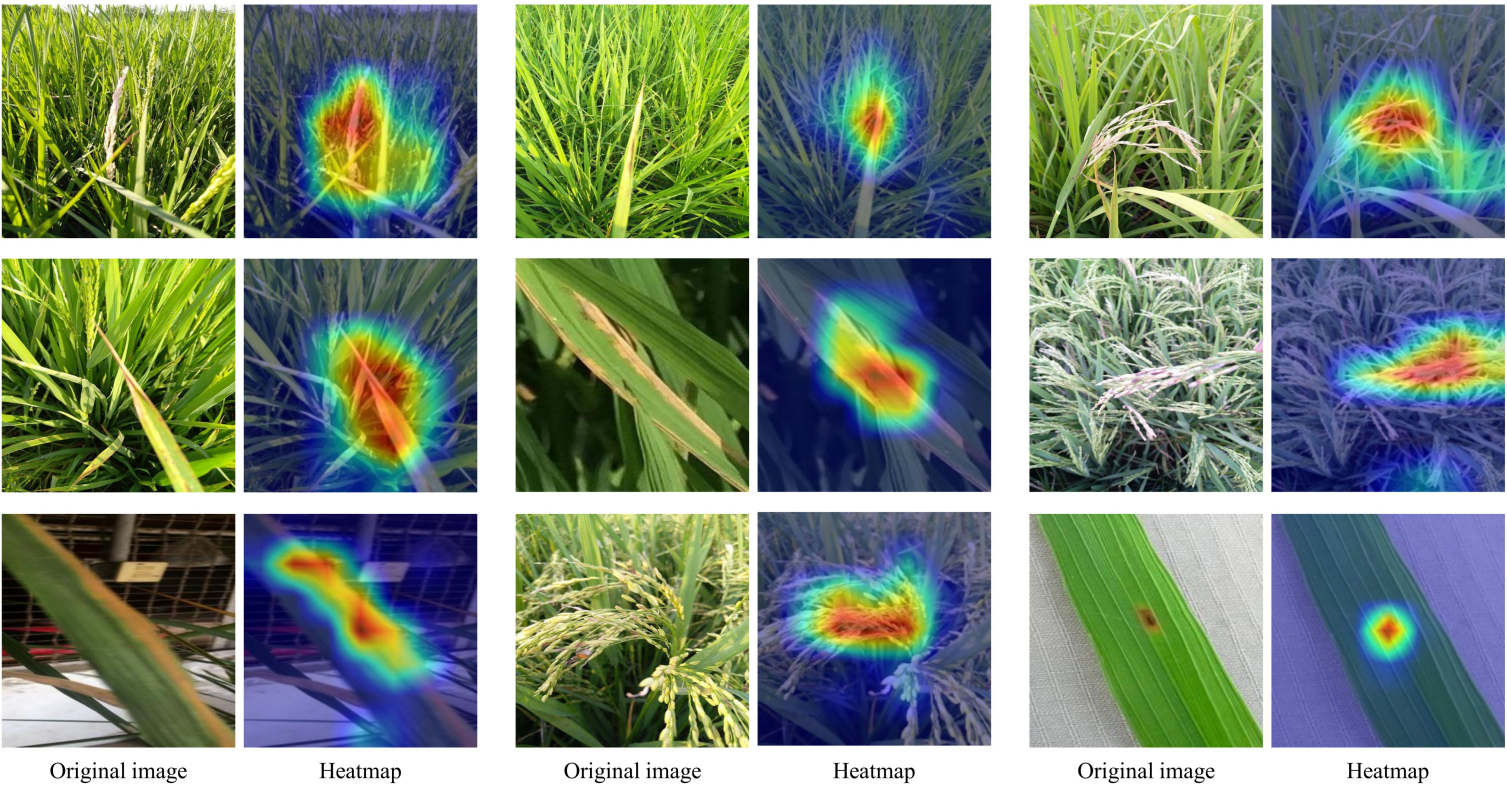
Visual presentation of the recognition results.

This study further employs Grad-CAM to visualize the decision-making basis of the proposed method in rice disease recognition, with the results shown in **Figure 10**. Grad-CAM calculates the contribution weights of a given class to the convolutional layer feature maps through gradient backpropagation, then fuses them via weighted summation and maps the results onto the original image, generating a heatmap that highlights the regions of interest for the model. As observed in **Figure 10**, the heatmaps consistently and accurately cover the lesion areas or abnormal leaf tissues across different types of disease images. For example, for typical diseases such as leaf brown spots and panicle lesions, the model can concentrate high-response regions on the actual disease sites rather than on healthy areas or background regions. This indicates that the model makes classification decisions based on the phenotypic characteristics of the diseases rather than relying on irrelevant noise or environmental information. Furthermore, by comparing the original images with the corresponding heatmaps, it is evident that the model demonstrates strong localization ability for diseases of varying scales and shapes, showing high robustness and generalization capability. This not only validates the method’s effectiveness in disease recognition but also enhances the interpretability of its decision-making process. Overall, the Grad-CAM visualization results demonstrate that the proposed method is able to focus on the key feature regions of rice diseases, thereby achieving accurate disease recognition and providing reliable support for practical agricultural applications.

### 3.6 Computational complexity

To verify the computational complexity of the model, this study uses the number of parameters, FLOPs, and model size to evaluate the lightweight structure, with the results shown in **Table 4**. As can be seen from the table, LightWaveNet exhibits extremely low levels in parameters, FLOPs, and model size, with only 0.28 M parameters, 0.02 G FLOPs, and a model size of 1.33 MB. Among all comparison methods, LightWaveNet has both the smallest parameter count and model size, while achieving the highest recognition accuracy. Compared with the well-performing lightweight model MobileNetV3, LightWaveNet reduces the number of parameters by approximately 93%, FLOPs by 90%, and model size by more than 90%. In addition, LightWaveNet’s accuracy is 3.14 percentage points higher than that of MobileNetV3. This indicates that LightWaveNet is not only extremely compact in resource usage but also able to maintain high recognition accuracy. Compared with the strong heavyweight model DenseNet201, LightWaveNet’s parameter count is only about 1.5% of the latter, its FLOPs are only 0.5%, and its model size is more than 50 times smaller. Despite this enormous disparity in complexity, LightWaveNet’s accuracy is still 1.45 percentage points higher than DenseNet201. For other heavyweight networks (such as ConvNeXt, ResNet101, and ViT), even under abundant computational resources, their accuracy still falls short of LightWaveNet, indicating that simply increasing network scale does not guarantee performance improvement. These results are consistent with those in **Figures 8**, **9**, indicating that LightWaveNet can effectively enhance inter-class separability while maintaining intra-class consistency during feature extraction, enabling the model to learn more stable and generalizable features even at a smaller scale. In contrast, some models with high computational cost or large numbers of parameters (such as GFNet and ViT) consume substantial computing resources but fail to achieve ideal feature representations, resulting in subpar performance. Overall, LightWaveNet not only achieves a lightweight design but also delivers more competitive recognition results. With extremely low resource consumption, LightWaveNet has significant application potential in resource-constrained scenarios such as embedded devices, mobile inference, and real-time processing.

**Table 4:** Computational complexity of different models.

Model	Parameters (M)	FLOPs (G)	Model size (MB)	Accuracy (%)
ConvNeXt	49.46	8.70	188.00	88.69
Densenet201	18.11	4.30	70.30	94.45
EfficientNet	20.19	2.90	77.80	92.71
GFNet	59.52	11.20	227.00	74.68
RepViT	22.41	4.60	86.50	84.17
ResNet101	42.52	7.90	162.00	90.70
ViM	25.43	0.06	97.10	73.92
ViT	85.81	17.60	327.00	63.49
ResMamba	13.76	1.60	52.50	87.06
DeiT-tiny	5.53	1.10	21.10	74.39
GhostNet	3.91	0.12	15.10	88.90
Mobilenetv2	2.24	0.30	8.76	91.63
Mobilenetv3	4.21	0.20	16.20	92.76
MobileViT	5.00	1.80	19.20	89.76
ShuffleNet	2.49	3.00	9.69	89.69
LightWaveNet	0.28	0.02	1.33	95.90

### 3.7 Inference speed analysis

**Figure 11:**
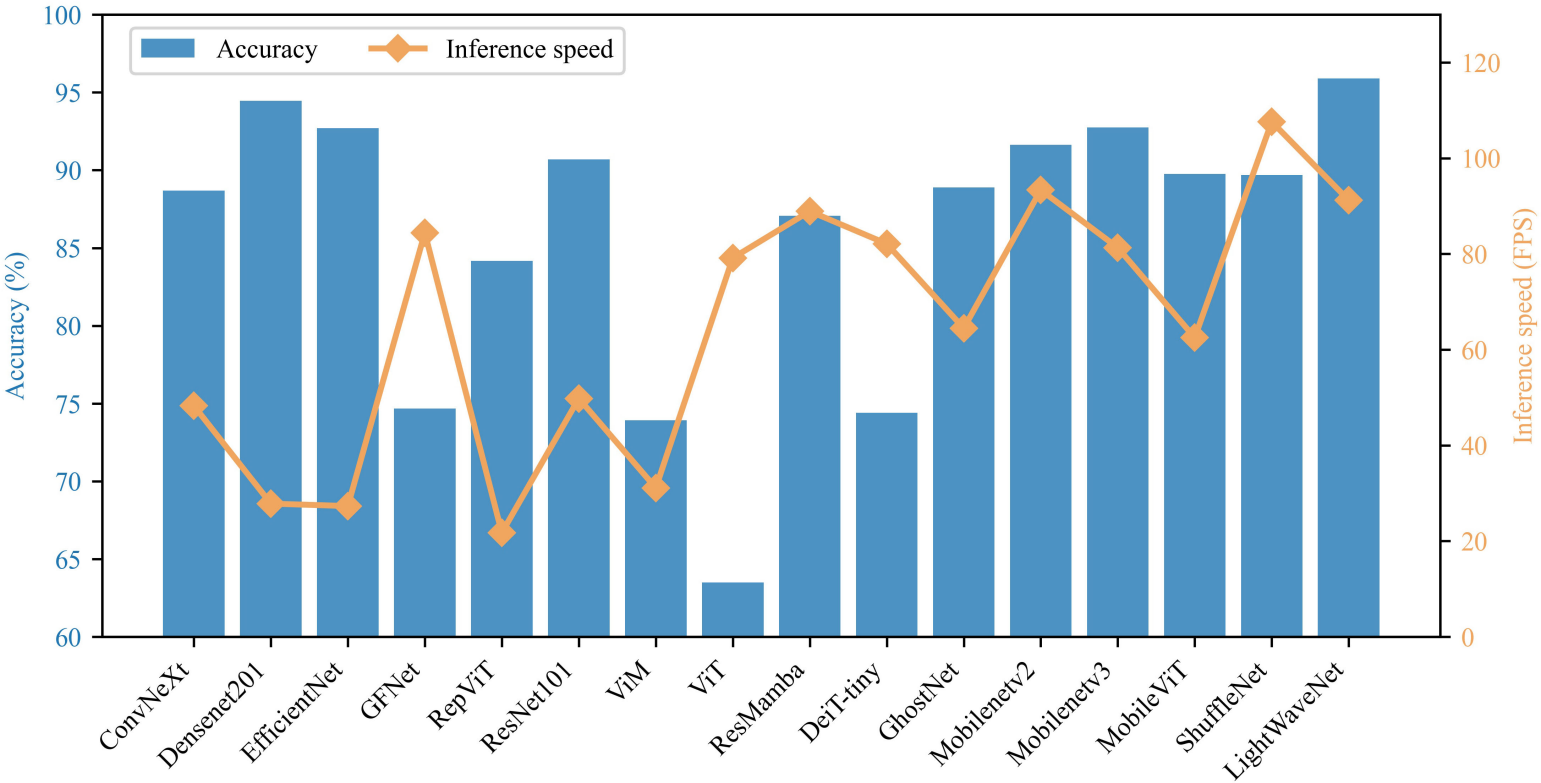
Comparison of different models in terms of accuracy and inference speed.

To evaluate the inference speed of the model, frames per second (FPS) was adopted as the evaluation metric, and the results are shown in **Figure 11**. As can be observed, most high-accuracy models (such as DenseNet201 and EfficientNet) perform well in terms of recognition accuracy, but their inference speed is relatively slow. Some lightweight models achieve higher FPS, but this is often accompanied by a notable performance drop, resulting in a trade-off characterized by high efficiency at the expense of low accuracy. In contrast, LightWaveNet demonstrates superior performance in both accuracy and FPS. It achieves higher classification accuracy than the comparison models while maintaining a relatively high inference speed. These results indicate that the proposed LightWaveNet successfully achieves an optimal balance between accuracy and efficiency, providing a promising solution for deployment in resource-constrained real-world application scenarios.

### 3.8 Robustness analysis

**Figure 12:**
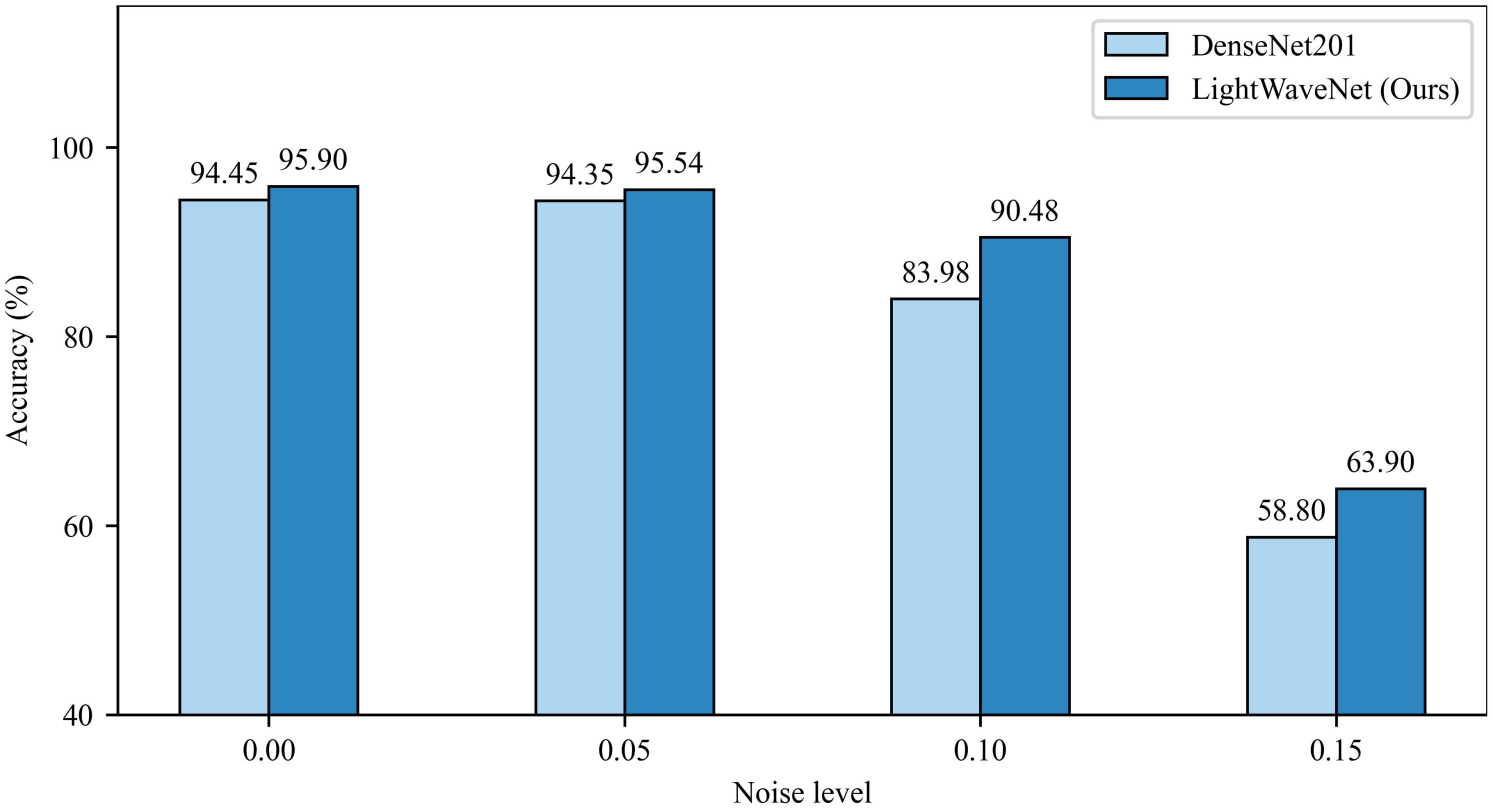
Comparison results under different Gaussian noise levels.

To evaluate the stability and reliability of LightWaveNet in complex and variable real farmland environments, a noise perturbation experiment was conducted. Specifically, different levels of Gaussian noise (Noise level = 0.00, 0.05, 0.10, 0.15) were added to the test set to simulate possible sensor noise or transmission distortion during real image acquisition. Meanwhile, the best-performing comparison model, DenseNet201, was selected as a baseline for comparison, and the results are shown in **Figure 12**. The results indicate that under noise-free and low-noise conditions (0.00 and 0.05), both models maintain high classification accuracy, while LightWaveNet consistently performs slightly better than DenseNet201, suggesting a certain performance advantage under standard scenarios. As the noise level increases to 0.10 and 0.15, the accuracy of DenseNet201 drops significantly, whereas the performance degradation of LightWaveNet is relatively smaller. In particular, LightWaveNet still maintains a clear advantage under high-noise conditions (e.g., 90.48% vs. 83.98% at 0.10, and 63.90% vs. 58.80% at 0.15). These results indicate that LightWaveNet is less sensitive to noise perturbations during feature extraction and representation, demonstrating stronger robustness, generalization ability, and anti-interference capability. This implies that when facing imaging noise that may occur in real acquisition scenarios, LightWaveNet can maintain more stable recognition performance and is therefore more suitable for agricultural disease recognition in complex environments.

## 4 Discussions

In this study, the proposed LightWaveNet demonstrates a good trade-off between complexity and accuracy. Specifically, the WP-Dense module achieves the complementarity between spatial details and global contours through the parallel operations of wavelet convolution and max pooling. Wavelet convolution can decompose the input features at multiple scales, separating the image signal into low-frequency and high-frequency components. The low-frequency part captures the overall shape and structure, while the high-frequency part retains details such as textures, edges, and lesions. Compared with traditional convolution, wavelet decomposition is more suitable for processing complex details in disease images, as different types of lesions often exhibit significant differences in texture and edge variations. Meanwhile, the parallel max pooling branch further enhances the response of discriminative regions, highlighting the salient features of the lesions. In addition, the multi-stage supervised learning mechanism introduced in the training phase of LightWaveNet also provides important support for improving the model’s performance. By introducing auxiliary classifiers in the first three stages, the model can obtain explicit optimization constraints in the shallow and intermediate stages, which not only accelerates the convergence speed of the network but also avoids the potential representation insufficiency that may occur when relying solely on deep features. Ablation experiment results show that this supervision method can effectively improve the feature representation ability of the model, enabling LightWaveNet to achieve classification accuracy comparable to or even better than mainstream methods while maintaining extremely low parameter and computation amounts. Overall, LightWaveNet is suitable for deployment on resource-constrained devices and delivers stable, reliable performance in rice disease recognition, providing new insights and technical support for intelligent agricultural diagnosis.

In recent years, a large number of studies have focused on the design of lightweight rice disease recognition models in an attempt to balance model accuracy and efficiency under limited computational resources. **Table 5** presents recent research on lightweight models for rice disease recognition. Compared with existing lightweight models, LightWaveNet has significant advantages in terms of parameters and FLOPs, with only 0.28 M parameters and 0.02 G FLOPs, far below most existing methods. For example, compared with DGLNet (Yang et al., 2023b), the number of parameters in LightWaveNet is reduced by approximately 48 times, and its computational cost is further reduced. Compared with BEiT (Chakrabarty et al., 2024), the number of parameters is reduced by about 43 times, and FLOPs are reduced by about 6 times. In contrast, the MobileViT-DAP method proposed by Zhang et al. (2025a) has lower computational complexity, with 0.75 M parameters and 0.23 G FLOPs. However, compared with the proposed LightWaveNet, LightWaveNet’s parameter count is only 37% of that of MobileViT-DAP, and its FLOPs are only 1/11 of MobileViT-DAP, fully demonstrating the effectiveness of the proposed method in lightweight design. In addition, compared with LWDN (Dheeraj and Chand, 2024), LightWaveNet reduces the parameter count by more than 5 times and decreases computation by nearly 300 times, highlighting the potential application value of the proposed method on resource-constrained platforms. Overall, although existing methods have achieved varying degrees of breakthroughs in lightweight design, most of them still contain significant redundancy in parameter scale or computational cost. In contrast, LightWaveNet, through the combination of WP-Dense and multi-stage supervised learning, achieves a balance between high accuracy and an extremely small model size. These results indicate that LightWaveNet not only has advantages in lightweight design but also provides a more feasible solution for the practical deployment of rice disease recognition models on mobile and edge devices.

**Table 5:** Comparison of model complexity between LightWaveNet and other lightweight methods for rice disease recognition.

Method	Year	Parameters (M)	FLOPs (G)	References
DGLNet	2023	13.50	0.21	Yang et al. (2023b)
BEiT	2024	12.00	0.12	Chakrabarty et al. (2024)
PMVT-S	2023	5.06	1.59	Li et al. (2023)
DepMulti-Net	2025	13.50	–	(Hu et al., 2025)
DCFM	2025	3.90	0.39	Qi et al. (2025)
DAL-Net	2025	3.60	–	Kang et al. (2025)
LWDN	2024	1.50	5.83	Dheeraj and Chand (2024)
CBAM-ParNeXt	2025	2.91	1.02	Lv et al. (2025)
AgriFusionNet	2025	12.00	–	Albahli (2025)
DC-GhostNet	2024	4.35	0.14	Peng et al. (2024)
LitePlantProto	2025	4.16	0.27	Wei et al. (2025)
MobileViT-DAP	2025	0.75	0.23	Zhang et al. (2025a)
LightWaveNet	–	0.28	0.02	Ours

Although LightWaveNet performs excellently in both accuracy and efficiency, it still has some limitations. For example, wavelet convolution is relatively sensitive to noise when decomposing high- and low-frequency components, which may lead to feature extraction deviations when the quality of input data is insufficient. To address this, future work could consider integrating adaptive wavelet decomposition strategies to mitigate the impact of noise, as well as exploring more efficient lightweight attention mechanisms to further enhance feature representation. In addition, although LightWaveNet greatly reduces computational complexity, excessive compression of the model size may limit its ability to adapt to more complex disease patterns and diverse environmental noise. Therefore, future research could explore combining the model with techniques such as knowledge distillation and dynamic inference to further enhance its representational capacity while maintaining extremely low complexity. From the perspective of data utilization, current experiments primarily emphasize image-level classification, while the integration of multi-modal information, such as temporal data and agronomic knowledge, is still limited. To address this issue, future studies could integrate hyperspectral imagery, meteorological data, and image information, enabling the model to develop a more comprehensive understanding of the mechanisms of disease occurrence. Despite certain limitations, the proposed LightWaveNet provides a valuable research foundation for intelligent rice disease recognition and offers useful insights for the application of lightweight deep learning models in smart agriculture.

## 5 Conclusions

In this study, we propose a lightweight rice disease recognition network, named LightWaveNet, which aims to reduce model complexity while maintaining recognition accuracy, making it suitable for resource-constrained devices in agricultural scenarios. To enhance the network’s representation capability in disease regions, it employs wavelet convolution to effectively decompose the input images into high- and low-frequency components, thereby capturing both fine-grained texture features and overall morphological information of disease spots. In addition, a multi-stage supervision mechanism is introduced during training, which constrains and guides features at different layers across various stages, improving the model’s convergence speed and stability. Experimental results show that, compared with other state-of-the-art lightweight algorithms, LightWaveNet achieves lower computational complexity and superior recognition accuracy. Ablation studies further validate the effectiveness of different modules within the network, demonstrating their critical role in recognizing various disease features. Overall, this study presents a new solution for efficient, accurate, and low-cost intelligent rice disease recognition, and serves as an important reference for smart agriculture, disease monitoring, and precision prevention and control.

## Data Availability

Publicly available datasets were analyzed in this study. This data can be found here: https://data.mendeley.com/datasets/fwcj7stb8r/1.

## References

[B1] Ahad M. T. Li Y. Song B. Bhuiyan T. (2023). Comparison of CNN-based deep learning architectures for rice diseases classification. Artif. Intell. Agric. 9, 22–35. doi: 10.1016/j.aiia.2023.07.001

[B2] Albahli S. (2025). AgriFusionNet: A lightweight deep learning model for multisource plant disease diagnosis. Agriculture 15, 1523. doi: 10.3390/agriculture15141523

[B3] Chakrabarty A. Ahmed S. T. Islam M. F. U. Aziz S. M. Maidin S. S. (2024). An interpretable fusion model integrating lightweight CNN and transformer architectures for rice leaf disease identification. Ecol. Inf. 82, 102718. doi: 10.1016/j.ecoinf.2024.102718

[B4] Chen Z. Cai Y. Liu Y. Liang Z. Chen H. Ma R. . (2025). Towards end-to-end rice row detection in paddy fields exploiting two-pathway instance segmentation. Comput. Electron. Agric. 231, 109963. doi: 10.1016/j.compag.2025.109963

[B5] Dheeraj A. Chand S. (2024). LWDN: lightweight DenseNet model for plant disease diagnosis. J. Plant Dis. Prot. 131, 1043–1059. doi: 10.1007/s41348-024-00915-z

[B6] Dosovitskiy A. Beyer L. Kolesnikov A. Weissenborn D. Zhai X. Unterthiner T. . (2021). “ An image is worth 16x16 words: Transformers for image recognition at scale,” in International Conference on Learning Representations (Vienna, Austria: OpenReview), 1–22.

[B7] Fan Z. Lu D. Liu M. Liu Z. Dong Q. Zou H. . (2025). Yolo-pdgt: A lightweight and efficient algorithm for unripe pomegranate detection and counting. Measurement 254, 117852. doi: 10.1016/j.measurement.2025.117852

[B8] Finder S. E. Amoyal R. Treister E. Freifeld O. (2025). “ Wavelet convolutions for large receptive fields,” in European Conference on Computer Vision (Milan, Italy: Springer), 363–380.

[B9] Gong L. Gao B. Sun Y. Zhang W. Lin G. Zhang Z. . (2024). preciseslam: Robust, real-time, lidar–inertial–ultrasonic tightly-coupled slam with ultraprecise positioning for plant factories. IEEE Trans. Ind. Inf. 20, 8818–8827. doi: 10.1109/TII.2024.3361092

[B10] Haikal A. L. A. Yudistira N. Ridok A. (2024). Comprehensive mixed-based data augmentation for detection of rice leaf disease in the wild. Crop Prot. 184, 106816. doi: 10.1016/j.cropro.2024.106816

[B11] Han K. Wang Y. Tian Q. Guo J. Xu C. Xu C. (2020). “ GhostNet: More features from cheap operations,” in Conference on Computer Vision and Pattern Recognition (Seattle, WA, USA: IEEE), 1577–1586.

[B12] He K. Zhang X. Ren S. Sun J. (2015). “ Deep residual learning for image recognition,” in Conference on Computer Vision and Pattern Recognition (Las Vegas, NV, USA: IEEE), 770–778.

[B13] Howard A. G. Sandler M. Chu G. Chen L.-C. Chen B. Tan M. . (2019). “ Searching for mobileNetV3,” in IEEE International Conference on Computer Vision (Seoul, Korea (South: IEEE), 1314–1324.

[B14] Hu K. Zheng X. Su X. Wu L. Liu Y. Deng Z. (2025). Identification of rice leaf disease based on DepMulti-Net. Front. Plant Sci. 16. doi: 10.3389/fpls.2025.1522487, PMID: 40212878 PMC11983642

[B15] Huang G. Liu Z. Weinberger K. Q. (2017). “ Densely connected convolutional networks,” in IEEE Conference on Computer Vision and Pattern Recognition (Honolulu, HI, USA: IEEE), 2261–2269.

[B16] Jin S. Cao Q. Li J. Wang X. Li J. Feng S. . (2025). Study on lightweight rice blast detection method based on improved YOLOv8. Pest Manage. Sci. 81, 4300–4313. doi: 10.1002/ps.8790, PMID: 40119571

[B17] Kang C. Jiao L. Liu K. Liu Z. Wang R. (2025). Fast rice plant disease recognition based on dual-attention-guided lightweight network. Agriculture 15, 1724. doi: 10.3390/agriculture15161724

[B18] Li G. Wang Y. Zhao Q. Yuan P. Chang B. (2023). PMVT: a lightweight vision transformer for plant disease identification on mobile devices. Front. Plant Sci. 14. doi: 10.3389/fpls.2023.1256773, PMID: 37822342 PMC10562605

[B19] Li H. Ruan C. Zhao J. Huang L. Dong Y. Huang W. . (2025a). Integrating high-frequency detail information for enhanced corn leaf disease recognition: A model utilizing fusion imagery. Eur. J. Agron. 164, 127489. doi: 10.1016/j.eja.2024.127489

[B20] Li Z. Zhang Y. Lu J. Wang Y. Zhao C. Wang W. . (2025b). Better inversion of rice nitrogen nutrition index at early panicle initiation stage using spectral features, texture features, and wavelet features based on UAV multispectral imagery. Eur. J. Agron. 168, 127654. doi: 10.1016/j.eja.2025.127654

[B21] Liu X. Li Q. Yin B. Yan H. Wang Y. (2024a). Assessment of macro, trace and toxic element intake from rice: differences between cultivars, pigmented and non-pigmented rice. Sci. Rep. 14, 1–13. doi: 10.1038/s41598-024-58411-1, PMID: 38710769 PMC11074271

[B22] Liu Z. Mao H. Wu C.-Y. Feichtenhofer C. Darrell T. Xie S. (2022). “ A ConvNet for the 2020s,” in Conference on Computer Vision and Pattern Recognition (New Orleans, LA, USA).

[B23] Liu Z. Zhou G. Zhu W. Chai Y. Li L. Wang Y.-f. . (2024b). Identification of rice disease under complex background based on PSOC-DRCNet. Expert Syst. Appl. 249, 123643. doi: 10.1016/j.eswa.2024.123643

[B24] Lv P. Xu H. Zhang Q. Shi L. Li H. Chen Y. . (2025). An improved lightweight ConvNeXt for rice classification. Alexandria Eng. J. 112, 84–97. doi: 10.1016/j.aej.2024.10.098

[B25] Lv P. Xu H. Zhang Y. Zhang Q. Pan Q. Qin Y. . (2024). An improved multi-scale feature extraction network for rice disease and pest recognition. Insects 15, 827. doi: 10.3390/insects15110827, PMID: 39590426 PMC11594787

[B26] Mehta S. Rastegari M. (2022). “ MobileViT: Light-weight, general-purpose, and mobile-friendly vision transformer,” in International Conference on Learning Representations (Virtual) ( OpenReview), 1–26.

[B27] Nalley L. Tsiboe F. Durand-Morat A. Shew A. Thoma G. (2016). Economic and environmental impact of rice blast pathogen (magnaporthe oryzae) alleviation in the United States. PloS One 11, e0167295. doi: 10.1371/journal.pone.0167295, PMID: 27907101 PMC5131998

[B28] Pan C. Wang S. Wang Y. Liu C. (2025). SSD-YOLO: a lightweight network for rice leaf disease detection. Front. Plant Sci. 16. doi: 10.3389/fpls.2025.1643096, PMID: 40901551 PMC12399686

[B29] Pan J. Wang T. Wu Q. (2022). RiceNet: A two stage machine learning method for rice disease identification. Biosyst. Eng. 225, 25–40. doi: 10.1016/j.biosystemseng.2022.11.007

[B30] Peng H. Yao L. Liu H. Peng S. He H. Xu H. . (2024). Different life cycles of rice pests’ images recognition based on adaptive lightweight DC-ghost module. Expert Syst. Appl. 255, 124645. doi: 10.1016/j.eswa.2024.124645

[B31] Qi Y. Liu T. Guo S. Wu P. Ma J. Yuan Q. . (2025). Hyperspectral imaging combined with a dual-channel feature fusion model for hierarchical detection of rice blast. Agriculture 15, 1673. doi: 10.3390/agriculture15151673

[B32] Quan S. Wang J. Jia Z. Xu Q. Yang M. (2024). Real-time field disease identification based on a lightweight model. Comput. Electron. Agric. 226, 109467. doi: 10.1016/j.compag.2024.109467

[B33] Rao Y. Zhao W. Zhu Z. Zhou J. Lu J. (2023). GFNet: Global filter networks for visual recognition. IEEE Trans. Pattern Anal. Mach. Intell. 45, 10960–10973. doi: 10.1109/TPAMI.2023.3263824, PMID: 37030707

[B34] Reek J. E. Lambers J. H. R. Perret E. Chin A. R. O. (2024). Seed classification with random forest models. Appl. Plant Sci. 12, e11596. doi: 10.1002/aps3.11596, PMID: 38912131 PMC11192156

[B35] Sandler M. Howard A. G. Zhu M. Zhmoginov A. Chen L.-C. (2018). “ MobileNetV2: Inverted residuals and linear bottlenecks,” in Conference on Computer Vision and Pattern Recognition (Salt Lake City, UT, USA: IEEE), 4510–4520.

[B36] Sethy P. K. Barpanda N. K. Rath A. K. Behera S. K. (2020). Deep feature based rice leaf disease identification using support vector machine. Comput. Electron. Agric. 175, 105527. doi: 10.1016/j.compag.2020.105527

[B37] Song H. Yan Y. Deng S. Jian C. Xiong J. (2024). Innovative lightweight deep learning architecture for enhanced rice pest identification. Physica Scripta 99, 096007. doi: 10.1088/1402-4896/ad69d5

[B38] Tan M. Le Q. V. (2019). “ EfficientNet: Rethinking model scaling for convolutional neural networks,” in International Conference on Machine Learning (Long Beach, California, USA: PMLR), 1–11.

[B39] Touvron H. Cord M. Douze M. Massa F. Sablayrolles A. Jegou H. (2021). “ Training data-efficient image transformers & distillation through attention,” in International Conference on Machine Learning (Vienna, Austria: PMLR), 10347–10357.

[B40] Wang A. Chen H. Lin Z. Han J. Ding G. (2024a). “ RepViT: Revisiting mobile cnn from vit perspective,” in IEEE Conference on Computer Vision and Pattern Recognition (Seattle, WA, USA: IEEE), 15909–15920.

[B41] Wang N. Wu Q. Gui Y. Hu Q. Li W. (2024b). Cross-modal segmentation network for winter wheat mapping in complex terrain using remote-sensing multi-temporal images and DEM data. Remote Sens. 16, 1775. doi: 10.3390/rs16101775

[B42] Wang T. Li J. Wu H.-N. Li C. Snoussi H. Wu Y. (2022). Reslnet: deep residual LSTM network with longer input for action recognition. Front. Comput. Sci. 16, 166334. doi: 10.1007/s11704-021-0236-9

[B43] Wang Z. Wei Y. Mu C. Zhang Y. Qiao X. (2025). Rice disease classification using a stacked ensemble of deep convolutional neural networks. Sustainability 17, 124. doi: 10.3390/su17010124

[B44] Wei L. Tang J. Chen J. Mukamakuza C. P. Zhang D. Zhang T. (2025). A lightweight few shot learning model for crop pest and disease identification. Artif. Intell. Rev. 58, 1–23. doi: 10.1007/s10462-025-11323-6

[B45] Yang L. Yu X. Zhang S. Long H. Zhang H. Xu S. . (2023a). GoogLeNet based on residual network and attention mechanism identification of rice leaf diseases. Comput. Electron. Agric. 204, 107543. doi: 10.1016/j.compag.2022.107543

[B46] Yang Y. Jiao G. Liu J. Zhao W. Zheng J. (2023b). A lightweight rice disease identification network based on attention mechanism and dynamic convolution. Ecol. Inf. 78, 102320. doi: 10.1016/j.ecoinf.2023.102320

[B47] Zeng W. He M. (2024). Rice disease segmentation method based on CBAM-CARAFE-DeepLabv3+. Crop Prot. 180, 106665. doi: 10.1016/j.cropro.2024.106665

[B48] Zhang M. Lin Z. Tang S. Lin C. Zhang L. Dong W. . (2025a). Dual-attention-enhanced MobileViT network: A lightweight model for rice disease identification in field-captured images. Agriculture 15, 571. doi: 10.3390/agriculture15060571

[B49] Zhang T. Bi Y. Du J. Zhu X. Gao X. (2022). Classification of desert grassland species based on a local-global feature enhancement network and uav hyperspectral remote sensing. Ecol. Inf. 72, 101852. doi: 10.1016/j.ecoinf.2022.101852

[B50] Zhang T. Xuan C. Cheng F. Tang Z. Gao X. Song Y. (2025b). CenterMamba: Enhancing semantic representation with center-scan mamba network for hyperspectral image classification. Expert Syst. Appl. 287, 127985. doi: 10.1016/j.eswa.2025.127985

[B51] Zhang T. Xuan C. Ma Y. Tang Z. Gao X. (2025c). An efficient and precise dynamic neighbor graph network for crop mapping using unmanned aerial vehicle hyperspectral imagery. Comput. Electron. Agric. 230, 109838. doi: 10.1016/j.compag.2024.109838

[B52] Zhang T. Xuan C. Tang Z. Gao X. Cheng F. Li Q. (2025d). ResMamba: A state–space model approach and benchmark dataset for precise forage identification in desert rangelands. Expert Syst. Appl. 280, 127411. doi: 10.1016/j.eswa.2025.127411

[B53] Zhang T. Xuan C. Tang Z. Gao X. Cheng F. Liu S. (2025e). Cross-domain adversarial learning for forage mapping and alpha-diversity assessment from UAV hyperspectral imagery in desert rangelands. Comput. Electron. Agric. 239, 111001. doi: 10.1016/j.compag.2025.111001

[B54] Zhang T. Xuan C. Tang Z. Gao X. Liu S. Song Y. . (2026). Cross-task collaborative learning for aboveground biomass estimation from UAV hyperspectral imagery in sample-scarce desert rangelands. Comput. Electron. Agric. 243, 111386. doi: 10.1016/j.compag.2025.111386

[B55] Zhang X. Zhou X. Lin M. Sun J. (2018). “ ShuffleNet: An extremely efficient convolutional neural network for mobile devices,” in IEEE Conference on Computer Vision and Pattern Recognition (Salt Lake City, UT, USA: IEEE), 6848–6856.

[B56] Zhao G. Zhao Q. Webber H. Johnen A. Rossi V. Junior A. F. N. (2024). Integrating machine learning and change detection for enhanced crop disease forecasting in rice farming: A multi-regional study. Eur. J. Agron. 160, 127317. doi: 10.1016/j.eja.2024.127317

[B57] Zhao T. Zhou H. Yan M. Zhou G. He C. Hu Y. . (2025). LVR: A language and vision fusion method for rice diseases segmentation under complex environment. Eur. J. Agron. 168, 127599. doi: 10.1016/j.eja.2025.127599

[B58] Zhu L. Liao B. Zhang Q. Wang X. Liu W. Wang X. (2024). “ Vision Mamba: Efficient visual representation learning with bidirectional state space model,” in International Conference on Machine Learning (Vienna, Austria: PMLR), 62429–62442.

